# Mining Centuries Old *In situ* Conserved Turkish Wheat Landraces for Grain Yield and Stripe Rust Resistance Genes

**DOI:** 10.3389/fgene.2016.00201

**Published:** 2016-11-18

**Authors:** Deepmala Sehgal, Susanne Dreisigacker, Savaş Belen, Ümran Küçüközdemir, Zafer Mert, Emel Özer, Alexey Morgounov

**Affiliations:** ^1^International Center for Maize and Wheat ImprovementTexcoco, Mexico; ^2^Crop Breeding Department, Transitional Zone Agricultural Research InstituteEskisehir, Turkey; ^3^Crop Breeding Department, Eastern Anatolia Agricultural Research InstituteErzurum, Turkey; ^4^Central Field Crops Research InstituteAnkara, Turkey; ^5^Crop Breeding Department, Bahri Dagdas International Agricultural Research InstituteKonya, Turkey; ^6^Crop Pathology Department, International Center for Maize and Wheat ImprovementAnkara, Turkey

**Keywords:** adult plant resistance, bread wheat, club wheat, genome wide association analysis, genotyping-by-sequencing

## Abstract

Wheat landraces in Turkey are an important genetic resource for wheat improvement. An exhaustive 5-year (2009–2014) effort made by the International Winter Wheat Improvement Programme (IWWIP), a cooperative program between the Ministry of Food, Agriculture and Livestock of Turkey, the International Center for Maize and Wheat Improvement (CIMMYT) and the International Center for Agricultural Research in the Dry Areas (ICARDA), led to the collection and documentation of around 2000 landrace populations from 55 provinces throughout Turkey. This study reports the genetic characterization of a subset of bread wheat landraces collected in 2010 from 11 diverse provinces using genotyping-by-sequencing (GBS) technology. The potential of this collection to identify loci determining grain yield and stripe rust resistance via genome-wide association (GWA) analysis was explored. A high genetic diversity (diversity index = 0.260) and a moderate population structure based on highly inherited spike traits was revealed in the panel. The linkage disequilibrium decayed at 10 cM across the whole genome and was slower as compared to other landrace collections. In addition to previously reported QTL, GWA analysis also identified new candidate genomic regions for stripe rust resistance, grain yield, and spike productivity components. New candidate genomic regions reflect the potential of this landrace collection to further increase genetic diversity in elite germplasm.

## Introduction

Wheat is an important crop in Turkey, planted on an area of more than 7 million ha with annual production exceeding 20 million tons (http://faostat.fao.org/). Consumption of bread and other wheat products in Turkey at more than 200 kg per capita is one of the highest in the world. The center of wheat origin and diversity is in the Fertile Crescent, a geographic area known as the Cradle of Civilization, which encompasses several countries in the Middle East and part of modern-day Turkey (Feldman, 2001). For this reason the diversity of wheat and its wild relatives in Turkey plays a global role as a significant genetic resource for wheat improvement (Karagöz, 2014). Wheat landraces are grown as small populations by farm communities in remote villages using centuries-old technologies, including hand planting and harvesting. The main reasons for the farmers to maintain these historic landraces are their adaptability to mountainous areas and excellent quality for home use.

IWWIP, which has been conducting research since 1986, is based in Turkey and develops germplasm for Central and West Asia. The main emphasis of IWWIP breeding is to improve broad adaptation, disease resistance and grain quality of winter wheat. More than 60 varieties originating from IWWIP have been released in Central and West Asia (http://old.cimmyt.org/en/where-we-work/asia/international-winter-wheat-improvement-program). In 2009, IWWIP initiated a Turkey national wheat landraces inventory for documentation, characterization, conservation, and utilization of landraces collected throughout the country. Within 5 years (2009–2014), around 2000 landrace collections from 55 provinces (from more than 1700 farmer's field) were documented and their diversity was described (Kan et al., 2015). IWWIP used the taxonomic (species) and botanical (morphotype) description approaches which were used by previous collectors in Turkey during 1920s and 1930s based on highly inherited spike traits: presence or absence of awns and their color; color and pubescence of spike glumes, spike density, and grain color (Zuev et al., 2013). In total, four species and 89 botanical varieties were collected during inventory (Morgounov et al., 2016). Like many other species in cradle areas of crop domestication, the phenotypic diversity of these wheat landraces is large and the source of endless treasure to breeders.

The unique feature of these landraces is their continuous cultivation by farmers without the introduction of scientific breeding. Over time, they have accumulated traits, which ensure their adaptation to changing climate, evolving agronomic practices and quality to meet the requirement of home use. Although landraces are commonly known to produce inferior yields to modern varieties under improved agricultural practices and are susceptible to diseases, they are also known to be more drought, cold, and heat tolerant. Disease resistance identified in landraces is likely to be more durable and novel. In a classic example, common bunt resistance in Turkish landraces was transferred to modern varieties, leading to millions of dollars in savings for the U.S. wheat industry (Bonman et al., 2006).

A detailed genetic characterization (assessment of genetic diversity, population structure, and dynamics of linkage disequilibrium across the genome) of this invaluable asset using molecular markers will not only allow leveraging of the full potential of this collection through association genetics but will also provide impetus to channelize the unexploited variation for prebreeding. Genotyping-by-sequencing (GBS) is rapidly becoming popular for low-cost high-density genome-wide scans through multiplexed sequencing (Poland and Rife, 2012). It has the advantage of providing a robust diversity estimate with a much reduced ascertainment bias in comparison to other whole-genome-genotyping technologies (Heslot et al., 2013) and it is therefore especially suited for genotyping genetic resources. The current study included bread wheat landraces collected in 2010 from 11 diverse provinces of Turkey for genetic evaluation. In order to validate the potential of this landrace collection for identifying loci determining quantitative and qualitative traits, we implemented genome wide association (GWA) analysis to: (a) dissect the genetics of grain yield (GY) and spike productivity components under different rainfed environments; (b) identify stable quantitative trait loci (QTL) related to these complex traits and for resistance to stripe rust; (c) study contribution of epistasis to the underlying genetic architecture of the traits; (d) exploit the genetic diversity in the collection.

## Materials and methods

### Association mapping panel

The landrace panel for the study was collected from central and southern parts of the Anatolian Plateau and the transitional zone between the plateau and the Mediterranean Sea (Supplementary Figure 1). In 2010, collection teams took spike samples from farmers' fields. After botanical description and classification the spikes were planted as head-rows in the fall of 2010 in Eskisehir and Konya (total ~2000 head rows) provinces. In 2011, selection of the best head rows took place while maintaining diversity. Selected head-rows were bulked and planted as an un-replicated trial in Konya in a plot of 4 m^2^ (total 450 lines). The superior 153 bread-wheat lines were selected based on grain yield and other agronomic traits under rainfed conditions and used in the 2012–2013 growing season for the current study (Supplementary Table 1). The selected lines belonged to bread wheat (*Triticum aestivum* ssp. *aestivum*), rare club wheat (*T. aestivum* ssp. *compactum*), which is no longer represented in modern varieties, and the intermediate *T. aestivum* ssp. *aestivum* grex *compactoidum* group.

### Phenotyping of the panel and statistical analysis

The landrace panel was phenotyped in three rain-fed environments typical of wheat production in Turkey (Supplementary Figure 1). Two of these environments are representative of the Central Anatolian Plateau at an altitude of around 1000 masl: Konya, in the south, has a semiarid climate (310–330 mm of rainfall a year) and cold winter (January minimum temperature: −4.7°C), whereas Eskişehir is slightly wetter (350–370 mm rainfall a year) and warmer (January minimum temperature: −3.6°C). The third site Erzurum in the East of Anatolian Plateau (1700 masl) represents an environment with the higher precipitation (570 mm) compared to Konya and Eskişehir with a longer and much colder winter (January minimum temperature: −9.7°C). All three experiments were conducted at research stations at the East Anatolian Agricultural Research Institute (Erzurum), Transitional Zone Agricultural Research Institute (Eskisehir), and Bahri Dagdas International Agricultural Research Institute (Konya). In each of the sites, there were a total of eight trials, each with a plot size of 7–8 m^2^ and with 25 entries in each trial (the remaining 47 entries were excluded from all analyses since they were durum wheat accessions). In all three environments no irrigation was provided. All trials were conducted in 2013 and each one utilized an alpha-lattice experimental design with at least two replications and two widely grown modern cultivars (Gerek and Karahan) as checks. Trials were grown using common agronomic practices: fallow as preceding crop; nitrogen fertilizer (N30) application and weed control in spring.

Agronomic traits viz., grain yield (GY), days to heading (DH), plant height (PH), spike length (SL), total and fertile number of spikelets per spike (TSS and FSPS, respectively), grain number per spike (GNPS), spike weight (SW), chaff weight per spike (CPS), grain weight per spike (GWPS), and thousand kernel weight (TKW) were measured in Eskisehir and Konya. At Erzurum only GY, DH, and PH was evaluated. Additional indices were calculated: spike density (number of spikelets per 10 cm); spike harvest index (% of grain in spike weight), spike fertility index (number of grains per 1 g of chaff). Days to heading was recorded as the number of days from January 1 until 50% of the plants of each plot reached heading stage. For evaluation of spike productivity 10 random spikes were collected from each plot and measured.

Stripe rust resistance was evaluated at adult-plant stage in the field under natural epidemic conditions in Erzurum and with artificial inoculation in Haymana and Izmir with a local pathogen population highly virulent on differentials carrying *Yr2, Yr6, Yr7, Yr8, Yr9, Yr25, Yr27, YrSd, YrSu*, and *YrA*. In addition, seedling reaction to stripe rust was independently evaluated in the greenhouse at Ankara using artificial inoculation with the same local population of the pathogens. Both field and greenhouse inoculation followed the internationally accepted methodology (McIntosh et al., 1995). Disease severity (scale 0–100% with 0 and 100 representing complete resistance and susceptibility, respectively) was recorded under the field conditions and classified as resistant (R; severity ≤ 20%), moderately resistant (MR; >20% up to 60%), moderately susceptible (MS; >60% up to 80%), and susceptible (S; >80%). For seedlings reaction, rust infection type (IT) on each genotype was recorded on day 18–22 based on a 0–9 scale with 0 and 9 representing complete resistance and susceptibility, respectively (Line and Qayoum, 1992). IT scores 0–3 were considered resistant, 4–5 as moderately resistant, 6–7 as moderately susceptible, and 8–9 as susceptible.

The descriptive statistics of each trait in each environment was calculated using the summary statistics option in GenStat 14th Edition. Data for each trait was checked for normality by drawing normal plots in GenStat. Broad sense heritability (*h*^2^) and adjusted means were calculated from a spatial model in the computer program ASReml-R (Butler et al., 2009). Pearson phenotypic correlation coefficients among traits were obtained in Minitab 15 (https://minitab.en.softonic.com/) for each environment. A yield stability coefficient was calculated for all entries using Lin and Binn's superiority index (Pi; Lin and Binns, 1988) in GenStat. The index uses the Pi parameters obtained by the following expression:

(1) $$Pi=\sum_{j\;=\;1}^{n}(X_{ij}-M_{j}{)}^{2}/2n$$

where *Pi* = superiority index of the i-th entry, *X*_*ij*_ = yield of the i-th entry in the j-th environment, *M*_*j*_ = maximum response obtained among all the entries in the j-th environment, and *n* = number of environments.

### Genotyping-by-sequencing

Genomic DNA was extracted from dried leaves collected from a bulk of 10 plants per accession using a modified CTAB (cetyltrimethylammonium bromide) method described in CIMMYT laboratory protocols (Dreisigacker et al., 2016) and quantified using NanoDrop 8000 spectrophotometer V 2.1.0. The genotyping of the landraces was accomplished using a GBS technique called DArTseq^™^ developed by DArT Pty. Ltd., Yarralumla, Australia. The detailed protocol is described in Sehgal et al. (2015). A consensus map version 3.0 developed by DArT Pty Ltd. (http://www.diversityarrays.com/sequence-maps) was provided as part of the service which contained ~64 K GBS markers and 4000 DArTs as anchored markers.

### Genotyping with stripe rust resistance genes linked markers

A total of 10 SSR/STS and SNP markers (LGC Genomics) linked to known stripe rust resistance genes were used for additional genotyping. These markers were linked to the genes *Yr29*/*Lr46, Yr30, Yr39, Yr41, Yr44, Yr50, Yr54*, and *Yr62* (Supplementary Table 2). PCR assays in single 10 μl reactions used to amplify SSR/STS primers contained final concentrations of 1x Buffer with Green Dye (Promega Corp., USA), 200 μM dNTPs, 1.5 mM MgCl_2_, 0.25 μM of each primer, 0.25U of DNA polymerase (GoTaq®;Flexi, Promega Corp., USA, Cat. # M8295), and 50 ng of DNA template. The PCR profile was 94°C for 2 min followed by 30 cycles of 94°C for 1 min, 54–60°C for 2 min (dependent on the primer), and 72°C for 2 min ending with 1 cycle of 72°C for 5 min and finally at 15°C. The amplified products were separated on 2–3% agarose gels in 1X TBE buffer. SNP markers were amplified using LGC Genomics KASP reagents (http://www.lgcgenomics.com) in reactions containing 2.5 ml water, 2.5 ml 2xKASPar Reaction mix, 0.07 ml assay mix, and 30 ng of dried DNA with a cycling program: 94°C for 15 min followed by 11 touchdown cycles of 94°C for 20 s, touchdown over 65–57°C for 60 s (dropping 0.8°C per cycle), 72°C for 30 s and followed by 26 cycles of 94°C for 20 s, 57°C for 60 s, and 72°C for 30 s, ending with 72°C for 2 min and final temperature at 20°C. Fluorescence was read as an end point reading at 25°C.

### Population structure

To estimate the number of subgroups in the panel, both principal components analysis (PCA) and model-based clustering via STRUCTURE 2.3.3 program (Pritchard et al., 2000) were used. SNPs having missing data <20% and minor allele frequency (MAF) ≥5% were included in both analyses. For STRUCTURE analysis, length of the burn-in period and the number of Markov Chain Monte Carlo replications were assigned at 50,000 with an admixture and allele frequencies correlated model. Three independent iterations were performed with the hypothetic number of subpopulation (K) ranging from 1 to 9. The correct estimation of K was provided by an *ad hoc* statistic ΔK (Evanno et al., 2005), based on the rate of change in the log probability of data between successive *K*-values. Based on the correct K, each landrace was assigned into a subpopulation for which the membership value (*Q*-value) was >0.5. The population structure matrix (Q) was generated for further analyses. For PCA analysis, the function PRCOMP was used from the STATS package in R (R Development Core Team, 2007) and 3D view of the PCA was drawn using an *in house* R script.

### Genetic diversity and linkage disequilibrium

Two diversity parameters, Nei's diversity index (DI; Nei, 1973) and polymorphic information content (PIC; Botstein et al., 1980), were calculated to characterize the genetic diversity of A, B, and D sub-genomes and that of different subpopulations using POPGENE version 1.32 (Yeh et al., 1997) and PowerMarker version 3.25 (Liu and Muse, 2005), respectively. Shannon's index for each of the subpopulations was calculated using POPGENE version 1.32. The linkage disequilibrium parameter (*r*^2^) for estimating the degree of LD between pair-wise SNPs was calculated using the software TASSEL 4.0 (http://www.maizegenetics.net) with 1000 permutations. The *r*^2^ values were then plotted against the genetic distance in centiMorgans to determine the pattern of LD decay across each chromosome, sub-genome and whole genome, respectively. The distance at which the LD decays to 0.1 was considered as the critical distance up to which a QTL region extends.

### Genome-wide association analysis

For GWA analysis, the compressed mixed linear model approach was utilized using the EMMAX package executed in R (Kang et al., 2010). The kinship matrix (*K*) generated with GAPIT package (Lipka et al., 2012) using the VanRaden algorithm (VanRaden, 2008) was used jointly with population structure (*Q* matrix/PC coordinates) to improve statistical power of association. For fixed effects, PCs through PCA analysis and *Q*-matrix through STRUCTURE were used as covariates. Scree plot of PCA revealed first three components as most informative and hence were used as covariate matrix in the *K* + *P* (kinship + PCA) model. For the *K* + *Q* (kinship + *Q*-matrix) model, a file generated with *K* = 7 in the STRUCTURE program was used as covariate matrix.

False discovery rate adjusted *p*-values generated using EMMAX package were very stringent. Therefore, to avoid potential risk of type II error, a threshold was determined by obtaining *p*-values within the bottom 0.1 percentile of the distribution. This approach has been used in a few recent GWA studies in wheat (Zegeye et al., 2014; Gao et al., 2016). Based on this approach, a threshold *p*-value of 0.001 and 0.0001 corresponded to the bottom 0.1 percentile of the distribution for grain yield and yield components and for stripe rust resistance, respectively. To declare significant MTA, these thresholds were combined with results of quantile-quantile (QQ) plots. For example, a marker was declared significant if it showed (a) *p*-value above the threshold and (b) deviation of its *p*-value from normal distribution curve in QQ plot. Adjacent co-segregating markers of a MTA were assigned to a unique QTL region upon meeting the following conditions: ≤ 10 cM of inter-marker genetic distance (based on average LD across genome) and presence of significant and strong LD among the markers (with *r*^2^ ≥ 0.5).

### Epistatic interactions

Epistatic interactions were determined among all markers which were declared to be significantly associated with grain yield and yield components and with stripe rust resistance. An *in house* R script was used which utilized a linear regression model to calculate *P*-values for two- and three-locus marker interactions. For all traits, genotypic data and PCA and kinship matrices were used. A significant threshold of *P* < 0.001 was used to declare significant marker-marker interactions. For stripe rust resistance, genes with frequency >0.5 were also used in determining epistatic interactions among them and the markers associated with APR to stripe rust.

### Stepwise regression

To discover the best allelic combination for higher yield across environments, a stepwise regression analysis (Moreno-Gonzalez, 1992) was conducted in GenStat 14th Edition. All possible combinations of significant markers were utilized and drawn against the average grain yield across environments.

## Results

### Wheat landraces performance for yield and yield components

Overall GY was highest in Erzurum province (3875 kg/ha) followed by Konya (2652 kg/ha) and Eskisehir (2463 kg/ha) reflecting the degree of moisture stress during the season. The three groups of landraces (bread wheat, *compactoidum* and club wheat) were fairly distinct in their spike structure, morphology and productivity components although the club and *compactoidum* group had more similarities. On an average, the *compactoidum* group was slightly higher yielding at the three sites compared to bread wheat (Supplementary Figure 2). Average GY of selected wheat landraces of the three groups in Erzurum and Konya was almost equal or slightly higher compared to the known Turkish variety Gerek, but inferior to the variety Karahan in all three sites (Supplementary Table 3). However, some of the top five highest yielding lines exceeded both checks in Erzurum and Konya but were inferior in Eskisehir. The selection from the landrace Albostan from Nevsehir province demonstrated yield potential exceeding 6 t/ha in Erzurum—33% higher than the highest yielding check Karahan. The Club wheat Comak selection from Aksaray province exceeded both checks under drought conditions in Konya. Bread wheat Elbistan and Akbugday from Aksaray, Goderedi club wheat from Karaman were almost as high yielding as checks in Eskisehir. These findings demonstrate the potential value of these top landrace selections as a genetic resource for breeding. The landraces PH exceeded 100 cm. They are slightly taller compared to the checks and also 3–4 days later in DH.

The analysis of yield components was largely based on spike productivity parameters in Eskisehir and Konya (Supplementary Table 4). The SL of *compactoidum* and club wheat was much shorter compared to bread wheat and checks. At the same time the TSS was slightly higher, resulting in much higher SD. As expected, the club wheat spike is short and dense, while the *compactoidum* group was intermedium but closer to club wheat than bread wheat. Interestingly, the percentage of fertile spikelets in these two groups was higher than bread wheat landraces and variety Gerek and equal to the high yielding check Karahan. On an average, the landraces with compact spikes had higher GNPS compared to bread wheat landraces (Supplementary Table 4). However, the bread wheat landraces had slightly larger grain with average TKW exceeding 40 g. Several bread wheat landraces like Elbistan from Aksaray and an unnamed *compactoidum* from Usak province had extremely large grain with TKW exceeding 50 g. The resulting trait, GWPS, had large variation with several landraces selections exceeding the Karahan variety.

The correlations between spike productivity characters and GY in Eskisehir and Konya provinces identified key traits contributing to higher yield under moisture stress in these two sites (Supplementary Table 4). In Eskisehir GNPS, GWPS, and TKW had the highest contributions to GY (*r* = 0.28; 0.27, and 0.24 respectively, *P* < 0.01). In Konya, the GNPS had the highest contribution to yield (*r* = 0.49) followed by GWPS (*r* = 0.46), GFS (*r* = 0.41), and number of FSPS (*r* = 0.32) (Supplementary Figure 3). Lack of strong association between these traits and GY indicates that different genotypes have a different pattern of spike productivity and yield formation and that none of the traits dominates in yield determination. The broad sense heritability (*h*^2^) estimates across environments were very high (≥0.90) for SL and SD and moderately high (≥0.60 to <0.90) for the remaining traits (Supplementary Table 5).

### Reaction to yellow rust

Stripe rust severity data from three sites suggests that Haymana had the highest disease pressure for *compactoidum* (96%) and club wheat (94%) in comparison to bread wheat (53%) landraces followed by Izmir and Erzurum (Supplementary Figure 2, Supplementary Table 6). The broad sense heritability across environments was 0.55. The value of correlation coefficients for severity between the sites was significant but below 0.4 suggesting substantial genotype × site interaction. Based on the reaction of genotypes with the known resistance the common genes providing protection at Haymana and Izmir were *Yr1, Yr5, Yr10, Yr15*, and *Yr26*. Additional effective genes in Izmir were *Yr24* and *Yr27*. Only 13 landraces lines (8.5%) demonstrated resistance across three environments with average severity below or equal 30%. Most of these lines (11) represented bread wheat (nine botanical varieties) and originated from seven provinces. Taking into consideration the seedling tests in the greenhouse, only two genotypes out of 13 aforementioned demonstrated a seedling-resistant reaction, suggesting the presence of major genes. The rest were susceptible during the seedling stage, but resistant as adult plants with a high likelihood of resistance being controlled by minor genes.

### Genetic diversity and population structure

A total of 7961 GBS SNP markers filtered from the entire set were used for studying genetic relationships among landrace accessions. Map positions were obtained for 5002 markers which were used to calculate diversity parameters for each chromosome and sub-genome (Supplementary Table 7). The B sub-genome had the highest number of mapped markers (2456; 49.1%), followed by the A (1957; 39.1%) and D sub-genomes (589; 11.7%). The mean diversity index (DI) and polymorphic information content (PIC) was 0.26 and 0.21, respectively, across genome.

**Figure 1:**
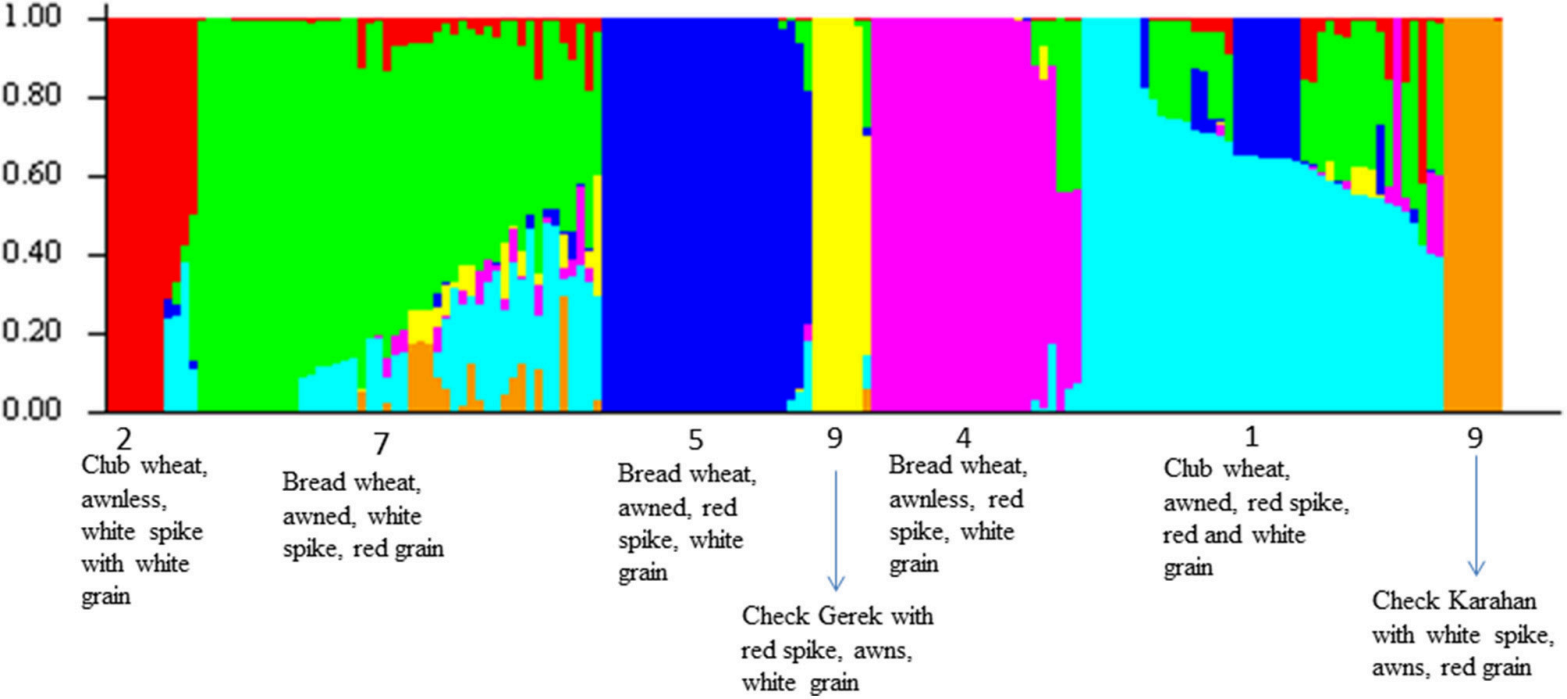
**Population structure of landraces based on 7961 GBS markers at *K* = 7**. Each accession is represented by a thin vertical line, which can be partitioned into seven colored segments representing estimated membership probabilities (Q) of the individual to the seven clusters. The numbers below the figure represent color + awn groups.

**Figure 2:**
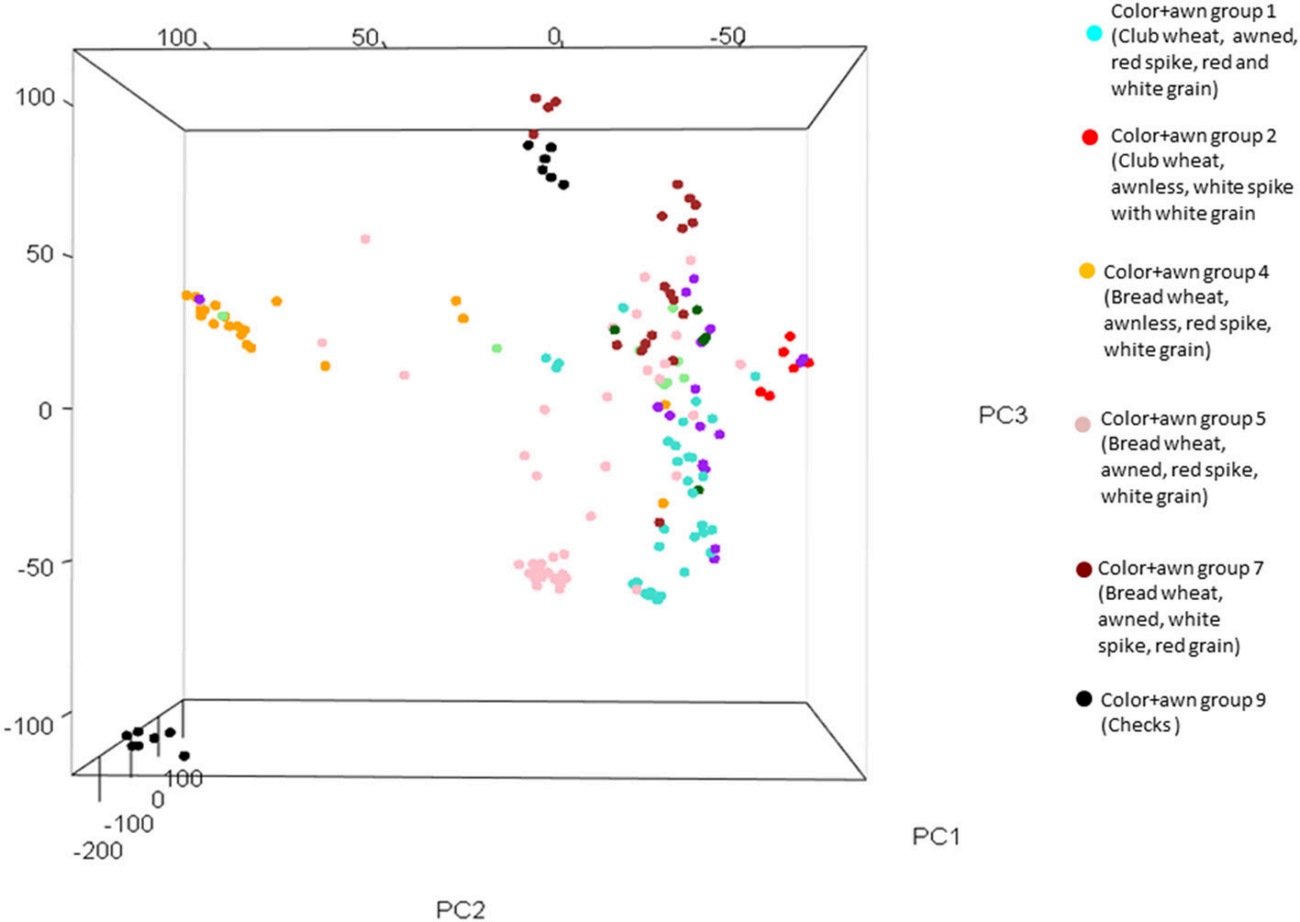
**Principal component analysis (PCA) plot with three principal axes showing six color+awn groups**.

For ascertaining the population genetic structure, results of STRUCTURE analysis were compared with PCA. At *K* = 2, the two subspecies, bread and club wheat (including *compactoidum*), differentiated (Supplementary Figure 4). The average gene diversity and Shannon's index within bread and club wheat individuals were 0.226 and 0.357 and 0.204 and 0.318, respectively. To identify the optimal number of K clusters, ΔK estimated with Evanno's method was plotted against the number of sub-groups K which revealed maximum value of ΔK at *K* = 7. The groups 1 (awned, red spike, red, and white grain) and 2 (awnless, white spike with white grain) clearly separated by the analysis were both *compactoidum* type. Groups 4 (awnless bread wheat, red spike, white grain), 5 (awned bread wheat, red spike, white grain), 7 (awned bread wheat with red grain), and 9 (checks) were differentiated at *K* = 7 (Figure 1). This structure was in partial accordance with the morphological differences between the landraces (Supplementary Table 8). The groups 3 (similar to group 7 but *compactoidum* type) and 6 (awnless bread wheat with white spike) with few entries and 8 (awned club wheat with different spike colors) were mixed with groups 1 and 7. The results of STRUCTURE analysis were confirmed using PCA plot. PC1, PC2, and PC3 accounted for 6.77, 4.89, and 4.12 % of the total variation, respectively (Figure 2, Supplementary Figure 5).

### Linkage disequilibrium

**Figure 3:**
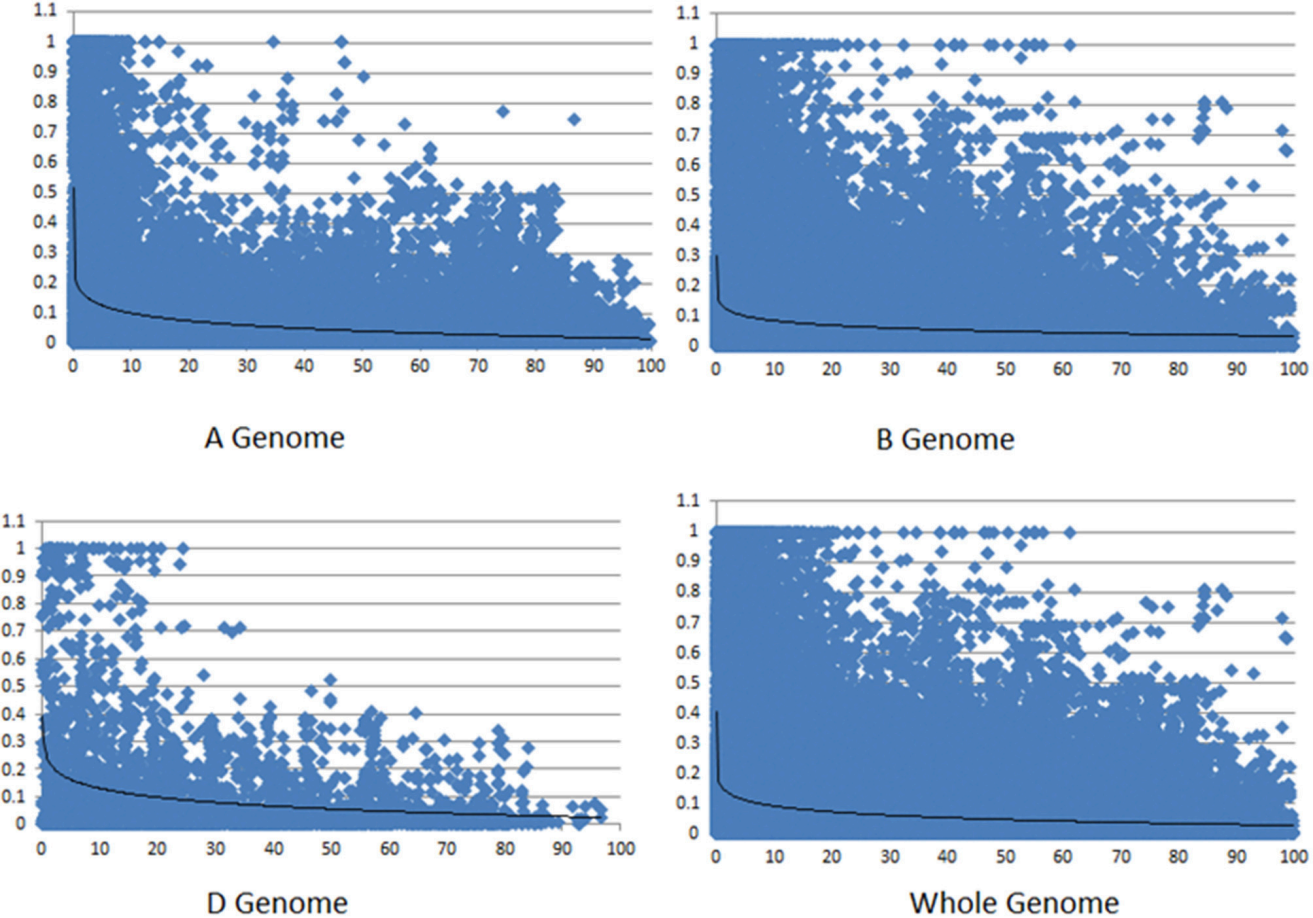
**LD decay across three sub-genomes and across whole genome**. Values at Y axis represent *r*^2^-values and values at X axis represents genetic distance in cM.

Linkage disequilibrium (LD) among markers and LD decay was calculated for all chromosomes (except chromosomes 4D and 5D each of which had insufficient number of markers to calculate LD). The highest LD was observed on the D sub-genome and both A and B sub-genomes showed lower and similar LD (Table 1). LD decayed below the baseline of *r*^2^ = 0.1 at about 15 cM for the A genome, while the smoothing curve crossed the *r*^2^ = 0.1 line at approximately 10 cM for the B genome (Figure 3). For the D genome, the curve crossed the baseline near 20 cM. For the whole genome, LD decay curve crossed the baseline at about 10 cM.

**Table 1:** **Summary of linkage disequilibrium across chromosomes and sub-genomes**.

Chromosome^*^	**Total number of marker pairs**	**% *r*^2^ > 0.1**	**Average LD at *r*^2^ > 0.1**	**LD decay below baseline *r*^2^ = 0.1 (in cM)**
1A	2926	16.6	0.25	2
1B	1378	17.4	0.29	5
1D	2415	25.1	0.35	20
2A	3570	20.2	0.30	8
2B	8001	15.8	0.30	5
2D	1081	27.2	0.36	25
3A	11175	15.7	0.32	2
3B	11476	14.4	0.28	5
3D	210	16.6	0.35	15
4A	3916	21.4	0.32	5
4B	2850	18.4	0.35	5
5A	8778	33.2	0.39	20
5B	18721	27.6	0.28	15
6A	9730	19.3	0.30	12
6B	50403	16.2	0.28	5
6D	351	14.8	0.36	12
7A	5886	14.2	0.27	5
7B	7140	26.0	0.35	25
7D	946	28.3	0.39	35
A genome	6569	20.0	0.30	15
B genome	14281	19.4	0.30	10
D genome	1001	22.4	0.38	20

* *LD and LD decay on chromosomes 4D and 5D were not calculated due to fewer markers*.

**Figure 4:**
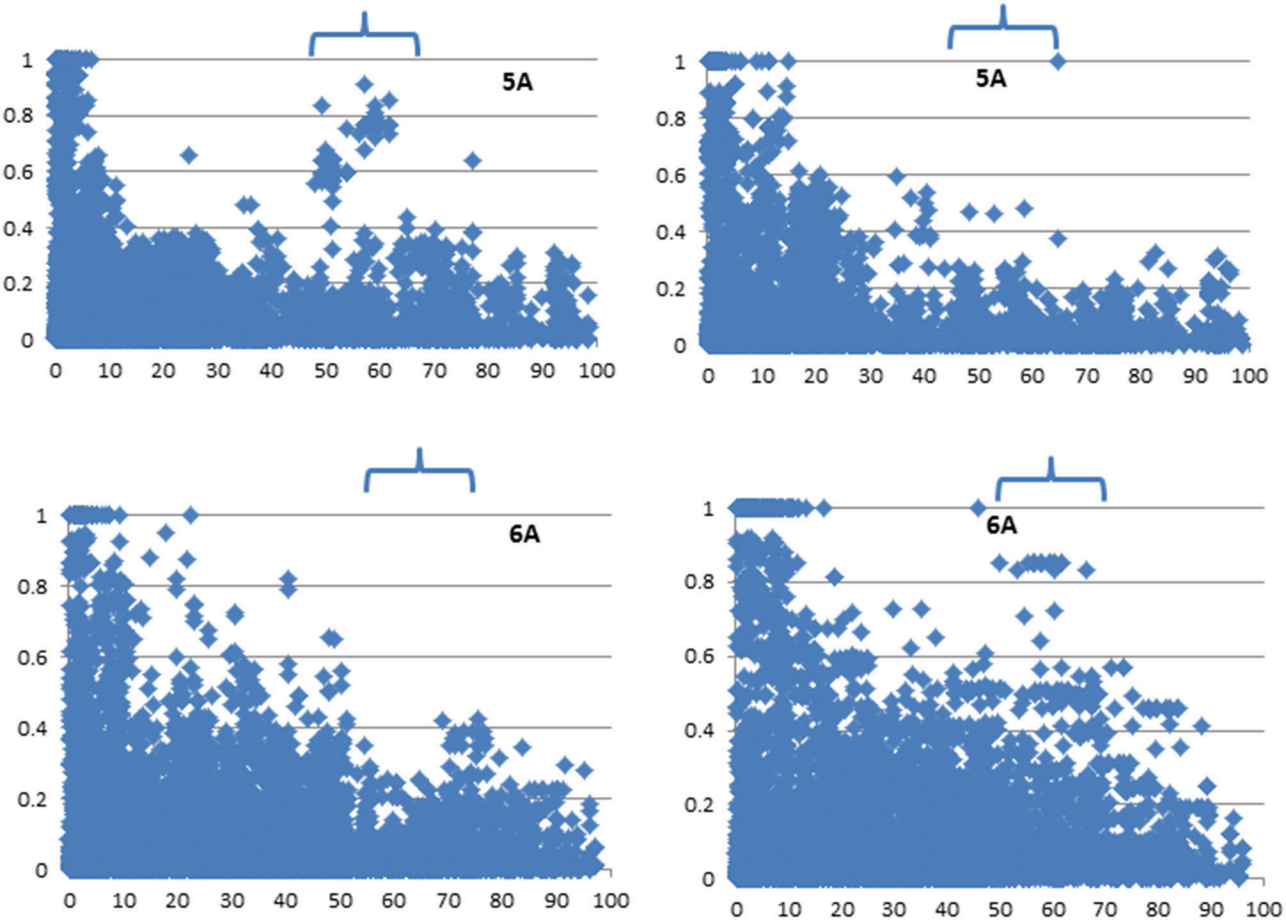
**Linkage disequilibrium among markers on 5A and 6A in bread (left)** and club wheat **(right)**. Values at Y axis represent squared correlation coefficient *r*^2^ and values at X-axis represent genetic distance in cM. The genomic regions are arrowed where the two subspecies show contrasting LD among markers.

The LD among markers was also calculated for all chromosomes in the two major subpopulations (bread and *compactoidum*/club wheat) and plotted against genetic distance which revealed relatively low *r*^2^ between adjacent marker pairs on some chromosomal areas of 2A, 2D, 3A, 4A, 4B, and 5A (Supplementary Figure 6, Figure 4) in club wheat. On chromosome 6A, a genomic region was identified where adjacent marker-pairs showed relatively low *r*^2^ in bread wheat (Figure 4).

### Marker-trait associations for grain yield and spike productivity characters

Marker-trait associations (MTA) were determined by two compressed mixed linear model analyses; (a) using K and Q matrices as covariates and (b) using K matrix and PCs as covariates. The results of two analyses revealed 99% similar results. Here, we report results using the second approach. GWA analysis was conducted for individual environments as well as for combined environments. QQ plots of combined environments analysis are presented in Supplementary Figure 7.

A total of 93 MTA controlling yield and spike productivity-associated traits were detected considering all environments (Supplementary Table 9). The number of MTA detected for GY in Konya was 12 which was higher than in both other environments, Eskisehir and Erzurum having 7 each. Considering all locations, a total of 24 MTA were obtained for GY distributed on 10 chromosomes viz. 1B, 2A, 2B, 3B, 4A, 4B, 5A, 5B, 6A, and 6B. Of these, six MTA identified on 1B, 2B, 5B, and 6A chromosomes [M3062 (1B; 177.18 cM), M3295 (2B; 119.56 cM), M2670 and M5746 (5B; 59.46 and 270.91 cM, respectively), M4457 (6A; 52.45 cM) and M6113 (12.36 cM)] were associated with GY in two out of three environments. Moreover, four out of the six markers (M3062, M3295, M2670, and M6113) also showed significant (*P* < *0.001*) association with yield stability coefficient (Supplementary Table 9). Stable MTA (detected in two and combined environments) were also detected for the spike parameters such as SL on chromosomes 1B, 6A, 6B, and 7B, SHI on chromosomes 1B and 6A, FI on chromosomes 1B, 5A, and 6B, TSS on chromosomes 2D and 5B and for TKW on chromosomes 2A and 3A. For DH, the most stable associations were detected on chromosome 2A at 113.0 and 231.7 cM (Supplementary Table 9) with an average percentage variation of 4.5 and 7.8%, respectively. MTA for PH were found on chromosomes 2A, 2B, 3B, 4B, 5B, and 6B, of which the ones on 2B, 3B, 4B, and 5B (one MTA on each of the chromosome) were the most consistent.

**Figure 5:**
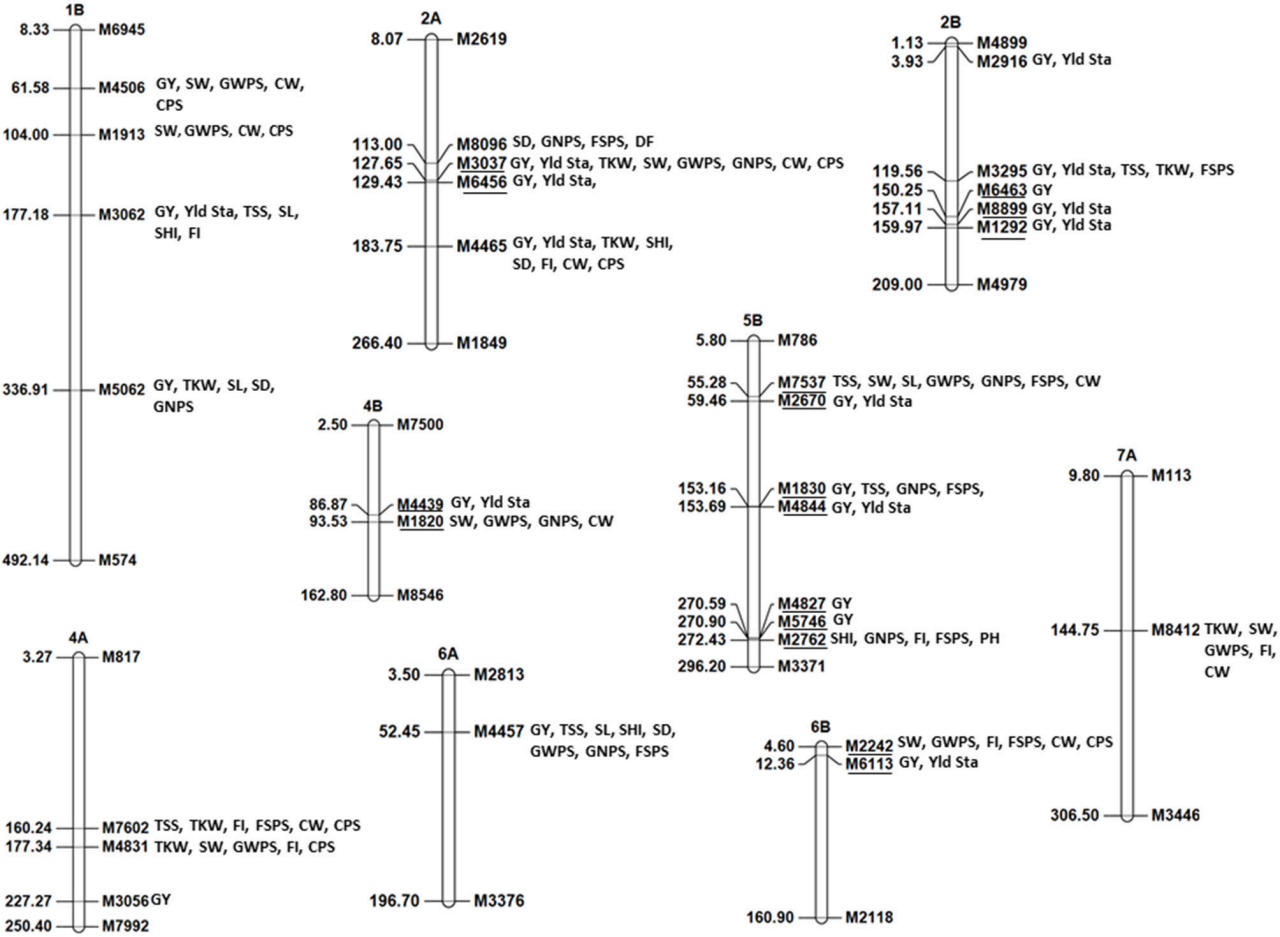
**Genomic regions associated with grain yield, yield stability, and multitraits (four or more than four traits) identified using 5003 GBS markers**. The underlined markers represent same genomic region.

Multi-trait MTA were detected in many chromosome regions (Figure 5). The most important genomic regions with MTA for four or more traits were detected on chromosomes 1B, 2A, 2B, 4A, 4B, 5B, 6A, 6B, and 7A (Figure 5). Of these, genomic regions on chromosomes 1B, 2A, 2B, 5B, and 6A were associated GY and spike productivity traits whereas genomic regions on chromosomes 4A, 4B, 6B, and 7A were associated mainly with spike productivity traits. Most importantly, the genomic region on 6A at 52.4 cM (M4457) was associated with GY in two environments and with seven spike traits including TSS, SL, SHI, SD, GWPS, GNPS, FSPS in at least one environment with percentage variation from 2 to 9% for different traits. The allele at this locus was associated with higher yield and a shorter spike length.

### Marker-trait associations for adult plant resistance to stripe rust

Due to the lack of phenotypic variation and the low frequency of seedling resistant lines observed in our panel, GWA analyses was only performed for adult plant resistance (APR) to stripe rust. Considering all three locations, 13 SNPs on seven chromosomes (1B, 2A, 2D, 3B, 4B, 5B, and 6B) were associated with stripe rust resistance at the adult plant stage (Table 2). Of these, 1, 4, and 3 SNPs were associated with resistance at Erzurum, Haymana, and Izmir environments, respectively, while remaining five SNPs were identified in either more than one environment or in combined environments analysis. A QQ plot of combined environment analysis is presented in Supplementary Figure 8. The SNP marker M6398 on chromosome 2AL at 159.4 cM was consistently detected at all three locations. The percentage of resistant to moderately resistant plants identified by the effective allele at each marker locus is shown in Table 2. Since the disease pressure was extremely high in Haymana (only 10.4% lines were resistant to moderately resistant), the percentage of resistant to moderate resistant plants identified at Haymana by the effective allele at each marker locus was the lowest at this location. A separate analysis was conducted for bread and club wheat individually which identified three loci exclusively in club wheat (Supplementary Table 10).

**Table 2:** **Markers associated with stripe rust response**.

**Marker**	**Chr**	**Pos (cM)**	**GBS clone ID**	**Erzurum**	**P_rE_**	**Haymana**	**P_rH_**	**Izmir**	**P_rI_**	**Combined environments**	Comment^***^
M6287^*^	1BL	261.7	1032134					0.06 (2 × 10^−4^)^**^	84.6		Novel
M6398^*^	2AL	159.4	1009953	0.05 (7 × 10^−5^)	90.0	0.04 (6 × 10^−4^)	40.0	0.03 (1 × 10^−4^)	64.0	0.04 (8 × 10^−4^)	Known (QRYr2A.2)
M8409	2AL	160.8	2279967			0.04 (8 × 10^−5^)	39.0				Known (QRYr2A.2)
M6660^*^	2DS	28.0	1697765	0.07 (1 × 10^−4^)	78.0			0.05 (3 × 10^−4^)	53.0	0.05 (3 × 10^−4^)	Novel
M1550^*^	2DL	164.6	991014	0.03 (4 × 10^−5^)	84.0						Known (QRYr2D.2)
M2676	3BL	133.4	1244829	0.09 (8 × 10^−6^)	82.0					0.04 (6 × 10^−5^)	Novel
M4676^*^	4BS	22.5	2266323			0.07 (3 × 10^−6^)	70.0				–
M6160^*^	4BS	59.8	991096					0.06 (3 × 10^−6^)	77.0		–
M3383	5BS	49.4	1862942	0.10 (6 × 10^−4^)	78.1					0.03 (9 × 10^−5^)	Known (QRYr5B.1)
M3486	5BS	79.8	988021					0.04 (8 × 10^−5^)	54.0		Novel
M7034	5BL	176.1	1194694			0.05 (4 × 10^−4^)	42.0				Novel
M6872^*^	5BL	232.3	2262945	0.08 (3 × 10^−5^)	83.0					0.04 (3 × 10^−4^)	Novel
M8217	6BS	60.7	2279771			0.03 (7 × 10^−4^)	38.0				Known (QRYr6BS.2)

*Chr, chromosome; Pos, position in cM*.

*P_rE_, P_rH_, and P_rI_ represent percentage of resistant to moderate resistant plants identified by the effective allele at each marker locus at Erzurum, Haymana, and Izmir, respectively*.

* *Markers involved in epistatic interactions. Underlined markers represent same QTL*.

** *R^2^-value of each marker and in parenthesis p-value*.

*** *Known QTL have been indicated as falling in metaQTL clusters reported by Rosewarne et al. (2013) based on 30 publications*.

We subsequently tested the segregation of eight stripe rust resistance genes previously published that were located in the observed MTA regions viz. *Yr29*/*Lr46, Yr30, Yr39, Yr41, Yr44, Yr50, Yr54*, and *Yr62* and investigated their possible association with stripe rust resistance in this panel. While the genes *Yr41* and *Yr30* were absent in the panel, resistant allele for the genes *Yr39, Yr44*, and *Yr54* was present in less than five percent of lines. Hence, markers linked with the latter genes were eliminated from association analysis due to their low frequency. The genes *Yr29*/*Lr46, Yr50*, and *Yr62* were segregating in the panel but only *Yr50* showed association with stripe rust response at the adult stage in Izmir environment.

### Epistatic interactions

Epistatic interactions were determined among main effect loci for grain yield and yield component traits which revealed that most of the traits were controlled by epistatic interactions (Supplementary Figure 9), except GNPS, GWPS, and FSPS for which main effects contributed more than epistatic effects. Supplementary Table 11 provides detail of key epicentric loci for the grain yield and yield traits in different environments.

Likewise, for stripe rust APR, epistatic interactions were observed among main effect loci at all field environments. Interestingly, the most stable marker (M6398) identified on 2AL (associated with APR to stripe rust in all environments) was also the main epicentric locus of epistatic interactions, which was interacting with markers on chromosomes 1BL, 2DS, 2DL, 4BS, and 5BL. When genes *Yr29*/*Lr46, Yr50*, and *Yr62* were included in the model, no interactions were observed (Supplementary Table 12).

### Stepwise regression analysis

**Figure 6:**
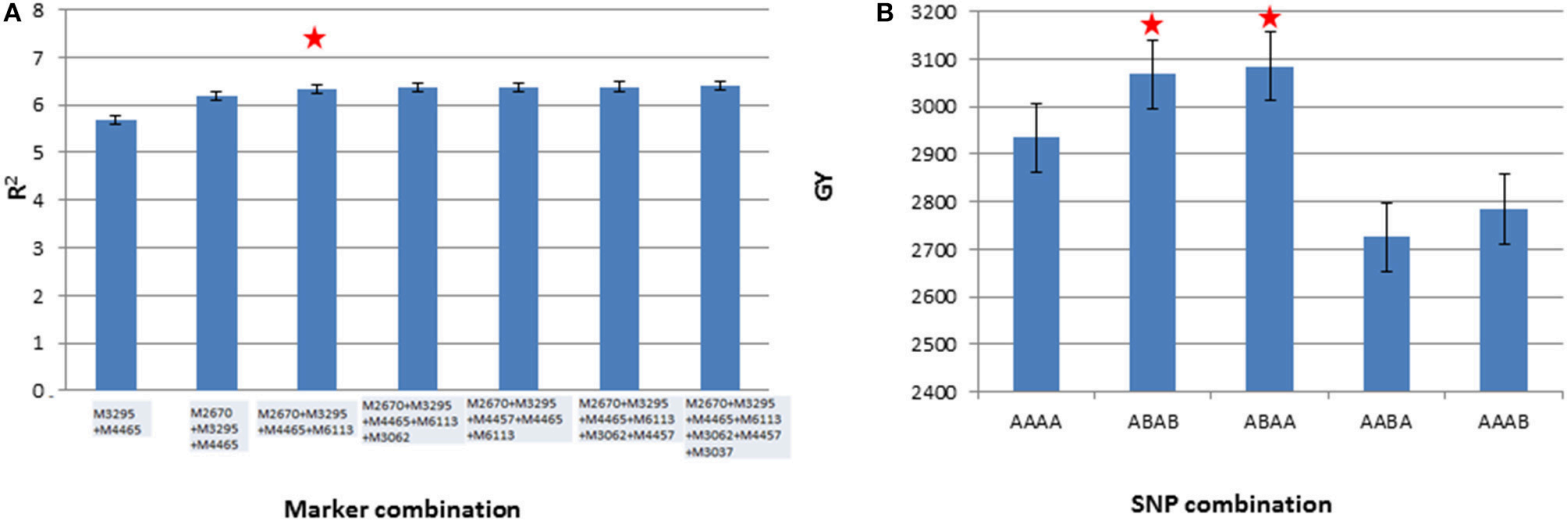
**Stepwise regression of the seven markers (A)** and average grain yield (GY) across three environments having 11 different combinations of four best markers identified through stepwise regression **(B)**. The starred column indicates the best marker and SNP combinations.

Seven markers (M3062, M3037, M4465, M3295, M2670, M4457, and M6113) from chromosomes 1B, 2A, 2B, 5B, 6A, and 6B were selected for the analysis. These markers were selected upon meeting at least one of the four following criteria: (1) showed association with GY in more than one environment, (2) showed association with yield stability coefficient, (3) showed association with spike productivity traits along with GY, and (4) showed epistatic interactions. The results of the analysis revealed that four marker combinations (M2670, M3295, M4465, and M6113), out of 33 combinations tried, resulted in the highest percentage variation for average grain yield across three environments (Figure 6A). For these four significant markers, two best allelic combinations are presented in Figure 6B.

## Discussion

The current study is the first report of assessment of genetic diversity, population structure, and dynamics of linkage disequilibrium across the genome of Turkish winter wheat landraces collected in 2010 from 11 diverse provinces within the country over 5 years (2009–2014) efforts made by IWWIP. Superior selections from the landraces have been identified with high yield at individual sites and across three locations, with high expression of spike productivity traits. The average diversity index (DI) of the landrace panel investigated in the study was 0.260; close to the value reported for landraces from Afghanistan and India (DI = 0.266 for both) but less than that of Iraq and Pakistan (DI = 0.340 and 0.300, respectively) using GBS markers (Sehgal et al., 2015). The panel was found to be structured broadly into two subpopulations; bread (*T. aestivum* ssp. *aestivum*; 64%) and *compactoidum*/club (*T. aestivum* ssp. *aestivum* grex *compactoidum* and *T. aestivum* ssp. *compactum*; 36%) wheat accessions. Similar genetic structuring was observed in a US Pacific Northwest winter wheat panel comprising bread and club wheat accessions (Naruoka et al., 2015). Accessions of bread wheat were genetically more diverse than that of club wheat probably due to its wider cultivation area and exposure to more diverse environments. Both subspecies were further substructured into red and white spike groups (Supplementary Figure 5), while the whole collection substructured into six groups depending on spike type and density, presence of awns and spike glume color. Significantly, no differentiation was observed based on province and/or any recorded agronomic trait as also was observed previously in a small landrace collection from Turkey (Dreisigacker et al., 2005). The old system of wheat diversity description used by Vavilov (Zuev et al., 2013) based on the similarity of highly inherited spike traits has accorded well with the genetic structuring of Turkish winter wheat landraces thus suggesting that spike morphology is one of the most important selection criterions adopted by farmers in Turkey.

Consistent with previous studies, we obtained fewer markers, highest levels of LD and the slowest LD decay for D sub-genome, which is suggestive of a lower frequency of effective recombinations due to a lower diversity of this sub-genome (Akhunov et al., 2010; Wang et al., 2014). The whole genome LD decay rate in the current study (~10 cM; Figure 3) is faster than previously published values in CIMMYT's Elite Spring Wheat materials (~30 cM; Crossa et al., 2007; Dreisigacker et al., 2012) and/or Chinese or European elite germplasm (~20–23 cM; Zhang et al., 2013; Nielsen et al., 2014). However, compared to other wheat global landrace collections (~4–5 cM; Neumann et al., 2011; Adhikari et al., 2012), LD decay rate of the present collection was slower. These results are not surprising considering that the diversities of the material used in the present and aforementioned studies vary extensively. Turkish landraces of the current study have been subjected to intense selection pressure by farmers over hundreds of years thus ensuring a slower LD decay. Characterization of differences in LD levels between chromosomes can also help to identify genomic regions subjected to genetic selection (Schlötterer, 2003). We identified such genomic regions on chromosomes 2A, 2D, 3A, 4A, 4B, 5A, and 6A in bread and *compactoidum*/club wheat, thus indicating that the landraces have been subjected to different selective pressures in these genomic regions probably due to different end uses of the two subspecies. Club wheat dominated production in Turkey in the 1920s (Gokgol, 1935). It was better suited for rainfed conditions and its grain being softer in texture was used for bulgur (cracked wheat normally boiled in water for consumption) rather than bread.

With the potential to exploit all recombination events occurring in the evolutionary history of a specific germplasm, GWA has become a powerful approach for dissection of complex agronomic traits in crops including bread wheat (reviewed in Sajjad et al., 2012). It has been suggested that MTA which are detected in more than one environment (stable MTA) or detected for multiple traits (pleiotropic MTA) prove beneficial for marker-assisted selection. Identifying pleiotropic MTA is particularly useful as yield components having higher correlation with GY can be used to enhance it. We obtained multi-trait genomic regions of grain yield and spike productivity components on nine chromosomes which resolved into 20 QTL (Figure 5). These multi-trait regions were compared with Meta QTL (MQTL) reported for grain yield and yield components by Zhang et al. (2010) and QTL published in studies thereafter (Supplementary Table 13). Briefly, out of the four QTL reported on chromosome 1B in the current study, the one at ~104 cM belongs to MQTL4 of Zhang et al. (2010). Some recent studies have also reported QTL for yield in this region under irrigated and drought stress/rainfed environments (Wu et al., 2012; Edae et al., 2014; Zhang et al., 2014). The remaining three QTL on chromosome 1B are novel of which the one identified at 177.1 cM is the most important, associated with yield stability across environments. For the three QTL reported on chromosome 2A, it was difficult to determine whether they fall in MQTL regions or were novel as flanking markers of only one of the MQTL, MQTL8, could be located on the consensus map.

On chromosomes 5B and 6B, no MQTL have been reported by Zhang et al. (2010). Some recent GWA studies have reported stable genomic regions associated with yield and yield related traits on these chromosomes under irrigated, drought, and/or rainfed conditions (Bordes et al., 2014; Edae et al., 2014; Ain et al., 2015; Lopes et al., 2015; Sukumaran et al., 2015). We obtained three genomic regions on 5B of which the one at 153.1–153.6 cM most likely falls in the region of the stable QTL for GY reported by Bordes et al. (2014). This genomic region was also identified to be stably associated with GY in diverse environments in a large elite panel of CIMMYT (Sehgal et al., under review). Similarly, the 7.7 cM (4.6–12.3 cM) QTL on 6B in the current study is ~13–15 cM from a stable QTL for GY identified by Bordes et al. (2014) on 6B using GWA approach. The QTL identified on chromosome 7A at 144.7 cM is ~22–32 cM away from reported MQTL (Zhang et al., 2010). In summary, various known QTL observed in advanced breeding lines could be detected in evolutionary earlier landrace accessions. In addition several new QTL were observed, emphasizing the importance of these landraces in further increasing the diversity of the breeding material.

The significance of epistasis in the genetics of yield traits has been demonstrated using bi-parental designs in many crop species (Xing et al., 2002; Ma et al., 2007) including wheat (Wu et al., 2012; reviewed in Goldringer et al., 1997). Using GWAM design in wheat, Reif et al. (2011) investigated the genetic architecture of grain yield and found that main effects dominated the control of grain yield and epistatic interactions contributed only a little. In contrast, our study has established that not only grain yield but also most yield associated traits were controlled by both main and epistatic effects (Supplementary Figure 9). This striking difference between the two studies was partly due to the types of AM populations used. Reif et al. (2011) used elite breeding lines in which fixation of alleles due to high selection intensity must have reduced epistasis among loci, whereas the current study included diverse landrace accessions in which fixation of QTL is less likely. Moreover, it has been suggested that complementary epistasis arise in environments with relatively low levels of resources (see Goldringer et al., 1997). Since Reif et al. (2011) used only irrigated environment, they noticed low level of epistasis for grain yield as compared to the current study involving three rainfed environments. Further, in the current study, the cumulative contribution from significant epistatic effects was relatively equal or higher to that from main effects for most traits except GNPS, GWPS, and FSPS (Supplementary Figure 9). These results are partly in congruence with Wu et al. (2012) who also reported higher contribution of main effects for GNPS and FSPS as compared to epistatic effects. For the remaining traits which were common with the current study (grain yield, thousand grain weight, total number of spikelets per spike, and spike length), the lower percentage of phenotypic variance explained by epistatic effects in their study could be attributed to detection of a large number of QTL with small effects.

GWA has been successfully deployed to identify markers associated with stem, leaf and stripe rust resistance in CIMMYT's historical bread wheat genotypes (Crossa et al., 2007) and for stem rust response in spring and winter wheat germplasm (Yu et al., 2011, 2012). However, reports showing MTA for stripe rust resistance in winter wheat germplasm using GWA are scanty (Naruoka et al., 2015). In the current study, six potentially novel QTL for resistance to stripe rust were identified in combined and individual environment analyses (Table 2) and one additional candidate novel QTL was detected exclusively in club wheat (Supplementary Table 10). The novel genomic region identified on 2DS at 28.0 cM is approximately 90–100 cM away from previously reported QTL for stripe rust response on 2DS using bi-parental populations (Bariana et al., 2001; Suenaga et al., 2003; Mallard et al., 2005; Melichar et al., 2008; Lu et al., 2009; Agenbag et al., 2012). Naruoka et al. (2015) recently reported a stable APR locus on 2DS (stable in nine environments) using GWA in a winter wheat association panel. However, their QTL is proximal to the QTL reported here. Similarly, the QTL on 3BL identified at 133.4 cM is ~25 and 50 cM away from stripe rust QTL clusters QRYr3B.2 and QRYr 3B.3, respectively, reported by Rosewarne et al. (2013). The third novel QTL identified in combined environments analysis on 5BL at 232.3 cM is ~ 88 cM away from QRYr5B.2 and QRYr5B.3 QTL clusters reported by Rosewarne et al. (2013).

Of the novel QTL identified in individual environment analyses, the one identified on 1BL at 261.7 cM was the most significant as it was suspected to be *Yr29*/*Lr46* (William et al., 2006). However, marker-marker association approach (Lopes et al., 2015) located *Yr29*/*Lr46* locus at the extreme distal end of the IBL (425.6 cM). Also, candidate gene based association mapping did not reveal any association of *Yr29* gene with stripe rust response at Izmir where the QTL was identified. Therefore, it is highly likely that this locus is potentially a new locus. Similarly, the locus on 2DL at 164.6 cM, which was recognized to belong to QTL cluster QRYr2D.2 of Rosewarne et al. (2013), was suspected to be APR gene *Yr54* (Basnet et al., 2015). We used *Yr54* gene-linked SSR marker to determine whether the two loci (M1550 and *Yr54*) are same or not. *Yr54* was segregating in our panel but the resistant allele was present in only 4 lines, while resistant allele of M1550 was present in 43% of lines, thus supporting the genetic distinctness of the two loci.

No stripe rust resistance gene has been reported on 4BS so far. An APR QTL has been reported in winter wheat on 4BS by Vazquez et al. (2012). The closely linked DArT (wPT-5265) marker of this QTL, however, is located on the long arm of the reference GBS consensus map. Hence, it was difficult to determine relative distance between the APR QTL obtained on 4BS in the current study and that of Vazquez et al. (2012). To emphasize again the value of our landrace collection, despite only a limited number of selections showed APR to yellow rust, many MTA are novel and will be worth introgressing into breeding material in the near future.

A wealth of information exist on epistatic interactions among APR loci for stem rust in wheat, even using GWAM designs (Kolmer et al., 2011; Yu et al., 2011, 2012; Haile et al., 2012; Singh et al., 2013). However, reports on epistatic interactions among APR loci for stripe rust in wheat are scarce (Hao et al., 2011; Vazquez et al., 2015). While Hao et al. (2011) did not observe any epistasis among the identified QTL in their study, Vazquez et al. (2015) reported significant epistatic interactions between QTL for stripe rust located on chromosomes 2AS and 6AL. Both abovementioned studies used bi-parental mapping populations. The current study is the first report of epistatic interactions among APR loci for stripe rust in a winter wheat germplasm using GWAM approach (Supplementary Table 12). The frequent involvement of 54% of main effect markers in significant epistatic interactions among them suggest that a network of gene-gene interactions is in part responsible for stripe rust resistance as also observed for stem rust resistance (Kolmer et al., 2011; Yu et al., 2011). It is noteworthy that the marker stably associated with stripe rust resistance on 2AL was also epistatically involved with other main effect loci in all environments. For combined environments, three main effect markers (M6872, M6660, and M6398) out of five were involved in epistatic interactions with percentage variation of up to 9.5% thus suggesting that they are critical to APR to stripe rust in this germplasm. Further, no epistatic interactions were observed between the stripe rust genes *Yr29*/*Lr46* and *Yr50* and any of the main effect markers which contrasts with the resistance mechanisms of e.g., the stem rust genes such as *Sr2* (Singh et al., 2009).

## Conclusions

We successfully identified both known associations (previously reported QTL) as well as new candidate genomic regions for both grain yield and spike productivity components and stripe rust resistance in the Turkish landrace panel. In total, eight and seven novel candidate genomic regions were identified for enhanced yield and stripe rust resistance, respectively. The co-incidence of the QTL identified in the current study in the same genomic regions as QTL reported in elite germplasms or other collections for grain yield and/or yield components points to the existence of “QTL hot spots” in wheat genome harboring QTL from diverse backgrounds. New candidate genomic regions reflect the potential of this landrace collection to increase genetic diversity in elite germplasm.

## Author contributions

DS generated gene-based marker data, performed diversity and association analyses and wrote the manuscript. SD generated GBS data. SD and AM designed the research and provided inputs in results and discussion. AM provided the germplasm for evaluation and coordinated field trials and subsequently provided phenotypic data on yield and spike characters. SB conducted field trial in Eskisehir and collected phenotypic data. UK conducted field trial in Erzurum and collected phenotypic data. ZM conducted field stripe rust evaluation in Haymana and collected disease data. EO conducted field trial in Konya and collected phenotypic data.

## Funding

The work was supported by funding from International Winter Wheat Improvement Program (IWWIP), Ministry of Food, Agriculture and Livestock of Turkish Republic and CGIAR/CRP WHEAT. Publication of this paper was supported by International Treaty on Plant Genetic Resources for Food and Agriculture project W2B-PR-41-TURKEY.

### Conflict of interest statement

The authors declare that the research was conducted in the absence of any commercial or financial relationships that could be construed as a potential conflict of interest.
