# Supplementary material for: Mining Centuries Old In situ Conserved Turkish Wheat Landraces for Grain Yield and Stripe Rust Resistance Genes
Source: Front Genet. 2016 Nov 18;7:201. doi: 10.3389/fgene.2016.00201 (PMC5114521; doi:10.3389/fgene.2016.00201)
Supplement: Supplementary file 2 [file Table2.DOCX]

| Gene | Marker type | Marker name | Reference |
| --- | --- | --- | --- |
| *Lr46* | SNP | Lr46_SNP1G22, CAPS csLv46 | Lagudah et al., personal communication |
| *Yr30/Sr2* | SNP | Sr2_ger9 3p | Mago et al., 2011 |
| *Yr41* | SSR | Xgwm410 | Luo et al., 2008 |
| *Yr39* | RGAP | Xwgp36, Xwgp45 | Lin et al., 2007 |
| *Yr54* | SSR | Xgwm301 | Basnet et al., 2014 |
| *Yr44* | SSR | Xgwm501 | Sui et al., 2009 |
| *Yr50* | SSR | Xgwm540 | Liu et al., 2013 |
| *Yr62* | SSR | Xgwm 192, Xgwm 251 | Lu et al., 2014 |

Supp. Table 2 Gene-based markers linked to stripe rust resistance genes used in the study
