# Supplementary material for: Mining Centuries Old In situ Conserved Turkish Wheat Landraces for Grain Yield and Stripe Rust Resistance Genes
Source: Front Genet. 2016 Nov 18;7:201. doi: 10.3389/fgene.2016.00201 (PMC5114521; doi:10.3389/fgene.2016.00201)
Supplement: Supplementary file 3 [file Table3.DOCX]

Supp. Table 3. Grain yield and other agronomic traits of selected wheat landraces and average for sub-species tested in three locations of Turkey, 2013

| Germplasm | | Average Grain Yield, kg/ha | | | | Plant height, cm | Days to heading | Stripe rust severity, % |  |
| --- | --- | --- | --- | --- | --- | --- | --- | --- | --- |
|  |  | Erzurum | Eskisehir | Konya | 3 sites | 3 sites^a^ | Konya | 3 sites^b^ |  |
| ***Gerek (check variety 1)*** | | ***3768*** | ***3256*** | ***2847*** | ***3290*** | ***89*** | ***128*** | ***39*** |  |
| ***Karahan (check variety 2)*** | | ***4643*** | ***3427*** | ***3861*** | ***3977*** | ***96*** | ***128*** | ***21*** |  |
| **Bread wheat landraces (*T. aestivum* sp. *aestivum*)** | | | | | | | | | |
| Elbistan (Aksaray) | | 4361 | 3162 | 3526 | 3683 | 104 | 133 | 83 |  |
| Kirmizi Agbugday (Aksaray) 86 | | 4472 | 2062 | 4157 | 3563 | 102 | 133 | 93 |  |
| Albostan (Nevsehir) | | 6181 | 2078 | 2262 | 3507 | 102 | 134 | 53 |  |
| Akbugday (Aksaray) | | 3847 | 3367 | 3113 | 3443 | 110 | 133 | 77 |  |
| Goderedi (Konya) | | 4660 | 2780 | 2876 | 3439 | 105 | 131 | 73 |  |
| ***Average (93 selections)*** | | ***3842*** | ***2408*** | ***2550*** | ***2937*** | ***103*** | ***131*** | ***62*** |  |
| **Bread wheat landraces (*T. aestivim* sp. *aestivum grex compactoidum*)** | | | | | | | | | |
| No name (Usak) | | 5458 | 2934 | 2890 | 3761 | 112 | 131 | 70 |  |
| Sahman (Aksaray) | | 5194 | 2572 | 3159 | 3642 | 103 | 134 | 62 |  |
| Sari Bugday (Konya) | | 4917 | 2608 | 2912 | 3479 | 103 | 134 | 67 |  |
| Kirmizi Bugday (Konya) | | 4965 | 2544 | 2890 | 3467 | 104 | 134 | 77 |  |
| Comak (Aksaray) | | 4104 | 2891 | 3391 | 3462 | 112 | 134 | 70 |  |
| ***Average (41 selection)*** | | ***3984*** | ***2590*** | ***2894*** | ***3156*** | ***102*** | ***132*** | ***68*** |  |
| **Club wheat landraces (*T. aestivum* sp. *compactum*)** | | | | | | | | | |
| Comak (Aksaray) | | 4403 | 2585 | 4058 | 3682 | 107 | 131 | 77 |  |
| Comak (Aksaray) | | 4451 | 2637 | 3325 | 3471 | 99 | 132 | 77 |  |
| Goderedi (Karaman) | | 4451 | 3022 | 2779 | 3417 | 98 | 129 | 77 |  |
| Koca Bugday (Kutahiya) | | 4736 | 2598 | 2394 | 3243 | 107 | 133 | 73 |  |
| Goderedi (Karaman) | | 4563 | 2878 | 2099 | 3180 | 101 | 129 | 47 |  |
| ***Average (17 selections)*** | | ***3919*** | ***2532*** | ***2764*** | ***3072*** | ***104*** | ***131*** | ***68*** |  |
|  | |  |  |  |  |  |  |  |  |
| Correlation with yield | Eskisehir | -0.17* | 1.00 | 0.14 | 0.50*** | 0.48*** | - | 0.17* |  |
|  | Konya | 0.03 | 0.14 | 1.00 | 0.62*** | 0.49*** | 0.17* | 0.25** |  |

^a^ – three sites: Erzurum, Eskisehir and Konya

^b^ – three sites: Erzurum, Hyamana and Izmir

*, **, *** coefficients of correlation significant at P<0.05; <0.01 and 0.001 respectively
