# Supplementary material for: Mining Centuries Old In situ Conserved Turkish Wheat Landraces for Grain Yield and Stripe Rust Resistance Genes
Source: Front Genet. 2016 Nov 18;7:201. doi: 10.3389/fgene.2016.00201 (PMC5114521; doi:10.3389/fgene.2016.00201)
Supplement: Supplementary file 4 [file Table4.DOCX]

Supp. Table 4. Grain yield components of selected wheat landraces and average for sub-species tested in Eskisehir and Konya, 2013

| Germplasm | | Spike length, cm | Spike density | Fertile spikelets per spike | % of fertile spikelets | Grains/  fertile spikelet | Chaff weight/ spike | Grain weight/ spike | Grains per spike | TKW |
| --- | --- | --- | --- | --- | --- | --- | --- | --- | --- | --- |
| ***Gerek (check variety 1)*** | | ***7.7*** | ***21.0*** | ***13.4*** | ***82.6*** | ***1.94*** | ***0.37*** | ***0.92*** | ***25.9*** | ***35.6*** |
| ***Karahan (check variety 2)*** | | ***9.4*** | ***18.7*** | ***15.9*** | ***86.7*** | ***1.87*** | ***0.47*** | ***1.06*** | ***28.4*** | ***37.4*** |
| **Bread wheat landraces (*Tr. aestivim* sp. *aestivum*)** | | | | | | | | | | |
| Elbistan (Aksaray) | | 8.6 | 20.3 | 14.0 | 80.0 | 1.37 | 0.46 | 1.40 | 19.2 | 52.5 |
| Kirmizi Agbugday (Aksaray) | | 8.8 | 19.4 | 14.3 | 84.3 | 1.79 | 0.32 | 1.02 | 25.7 | 37.6 |
| Albostan (Nevsehir) | | 9.3 | 19.2 | 13.5 | 75.7 | 1.40 | 0.41 | 1.17 | 18.8 | 33.3 |
| Akbugday (Aksaray) | | 8.0 | 21.6 | 14.3 | 82.7 | 1.42 | 0.42 | 1.29 | 20.3 | 36.5 |
| Goderedi (Konya) | | 9.1 | 18.2 | 13.2 | 79.8 | 1.75 | 0.29 | 0.98 | 23.0 | 45.3 |
| ***Average (93 selections)*** | | ***8.8*** | ***19.1*** | ***13.5*** | ***81.1*** | ***1.60*** | ***0.35*** | ***0.99*** | ***21.5*** | ***40.4*** |
| **Bread wheat landraces (*Tr. aestivim* sp. *aestivum grex compactoidum*)** | | | | | | | | | | |
| No name (Usak) | | 6.8 | 24.0 | 14.0 | 85.7 | 1.98 | 0.29 | 0.72 | 27.7 | 50.6 |
| Sahman (Aksaray) | | 5.6 | 31.2 | 15.3 | 88.5 | 1.79 | 0.31 | 0.96 | 27.5 | 37.1 |
| Sari Bugday (Konya) | | 6.1 | 30.6 | 16.3 | 87.5 | 1.85 | 0.27 | 0.63 | 30.2 | 39.0 |
| Kirmizi Bugday (Konya) | | 6.6 | 26.1 | 15.3 | 89.3 | 1.96 | 0.27 | 0.74 | 30.0 | 42.9 |
| Comak (Aksaray) | | 6.5 | 26.6 | 15.0 | 87.4 | 1.70 | 0.40 | 1.04 | 25.5 | 38.4 |
| ***Average (41 selection)*** | | ***5.8*** | ***29.5*** | ***14.6*** | ***86.5*** | ***1.77*** | ***0.35*** | ***0.87*** | ***25.8*** | ***38.3*** |
| **Club wheat landraces (*Tr. aestivim* sp. *compactum*)** | | | | | | | | | | |
| Comak (Aksaray) | | 5.1 | 34,9 | 15.5 | 89.4 | 1.80 | 0.33 | 1.17 | 27.8 | 42.0 |
| Comak (Aksaray) | | 5.0 | 38.0 | 17.0 | 90.3 | 1.80 | 0.31 | 1.10 | 30.7 | 36.0 |
| Goderedi (Karaman) | | 5.5 | 31.0 | 14.7 | 86.3 | 1.73 | 0.28 | 0.94 | 25.3 | 38.8 |
| Koca Bugday (Kutahiya) | | 5.1 | 32.6 | 13.7 | 82.8 | 1.65 | 0.32 | 0.80 | 22.5 | 35.7 |
| Goderedi (Karaman) | | 4.6 | 35.4 | 12.8 | 78.6 | 1.79 | 0.24 | 0.76 | 23.0 | 33.5 |
| ***Average (17 selections)*** | | ***5.1*** | ***33.5*** | ***14.4*** | ***85.7*** | ***1.71*** | ***0.29*** | ***0.90*** | ***24.6*** | ***36.5*** |
|  | |  |  |  |  |  |  |  |  |  |
| Correlation with yield | Eskisehir | -0.10 | 0.04 | 0 | 0.19* | 0.28** | 0.11 | 0.27** | 0.17* | 0.24** |
|  | Konya | -0.12 | 0.19* | 0.32*** | 0.24** | 0.41*** | 0.28** | 0.46*** | 0.49*** | 0.12 |

*, **, *** coefficients of correlation significant at P<0.05; <0.01 and 0.001 respectively
