# Supplementary material for: Mining Centuries Old In situ Conserved Turkish Wheat Landraces for Grain Yield and Stripe Rust Resistance Genes
Source: Front Genet. 2016 Nov 18;7:201. doi: 10.3389/fgene.2016.00201 (PMC5114521; doi:10.3389/fgene.2016.00201)
Supplement: Supplementary file 5 [file Table5.DOCX]

| Trait | Environment | Mean | SD | Min | Max | h^2^ |
| --- | --- | --- | --- | --- | --- | --- |
| GY | Erzurum | 3875.28 | 640.49 | 2465.00 | 5458.00 | 0.632213 |
|  | Hamidiye | 2463.02 | 515.73 | 1155.00 | 3587.00 |  |
|  | I.CUMRA | 2652.08 | 493.89 | 1553.00 | 3690.00 |  |
| DF | Erzurum | 154.55 | 4.28 | 146.00 | 164.00 | 0.714894 |
|  | I.CUMRA | 131.61 | 2.21 | 125.00 | 135.00 |  |
| PH | Erzurum | 103.33 | 8.66 | 80.00 | 125.00 | 0.747104 |
|  | Hamidiye | 107.99 | 6.76 | 92.00 | 125.00 |  |
|  | I.CUMRA | 97.78 | 8.09 | 79.00 | 118.00 |  |
| TSPS | Hamidiye | 17.48 | 1.31 | 14.33 | 20.67 | 0.742402 |
|  | I.CUMRA | 16.13 | 1.26 | 13.67 | 19.33 |  |
| TKW | Hamidiye | 51.11 | 7.04 | 34.93 | 65.26 | 0.863827 |
|  | I.CUMRA | 57.15 | 6.78 | 41.50 | 73.85 |  |
| SW | Hamidiye | 1.28 | 0.28 | 0.69 | 2.03 | 0.828526 |
|  | I.CUMRA | 1.18 | 0.24 | 0.59 | 1.75 |  |
| SL | Hamidiye | 7.79 | 1.99 | 4.10 | 10.87 | 0.935941 |
|  | I.CUMRA | 7.31 | 1.67 | 4.23 | 10.27 |  |
| SHI | Hamidiye | 72.24 | 2.81 | 65.70 | 79.04 | 0.813065 |
|  | I.CUMRA | 72.84 | 2.64 | 66.28 | 79.72 |  |
| SD | Hamidiye | 24.08 | 7.11 | 16.20 | 42.64 | 0.947783 |
|  | I.CUMRA | 23.16 | 5.92 | 12.91 | 39.06 |  |
| GWPS | Hamidiye | 0.92 | 0.21 | 0.49 | 1.49 | 0.832998 |
|  | I.CUMRA | 0.87 | 0.20 | 0.36 | 1.39 |  |
| GNPS | Hamidiye | 24.98 | 4.09 | 15.33 | 34.33 | 0.800243 |
|  | I.CUMRA | 21.05 | 4.04 | 10.67 | 30.33 |  |
| FS | Hamidiye | 14.87 | 1.65 | 11.00 | 19.00 | 0.784865 |
|  | I.CUMRA | 12.92 | 1.60 | 8.67 | 17.33 |  |
| FI | Hamidiye | 72.34 | 13.46 | 41.80 | 109.68 | 0.863272 |
|  | I.CUMRA | 65.76 | 12.14 | 35.37 | 98.44 |  |
| CW | Hamidiye | 0.35 | 0.07 | 0.19 | 0.55 | 0.820189 |
|  | I.CUMRA | 0.32 | 0.06 | 0.20 | 0.47 |  |
| CPS | Hamidiye | 0.02 | 0.00 | 0.01 | 0.04 | 0.856688 |
|  | I.CUMRA | 0.02 | 0.00 | 0.01 | 0.04 |  |

Supp. Table 5 Descriptive statistics and broad-sense heritabilities of grain yield and its components across environments
