# Supplementary material for: Mining Centuries Old In situ Conserved Turkish Wheat Landraces for Grain Yield and Stripe Rust Resistance Genes
Source: Front Genet. 2016 Nov 18;7:201. doi: 10.3389/fgene.2016.00201 (PMC5114521; doi:10.3389/fgene.2016.00201)
Supplement: Supplementary file 6 [file Table6.DOCX]

Supp. Table 6. Reaction of selected wheat landraces and average for sub-species to stripe rust at three sites of Turkey

| Germplasm | | Botanical variety | Stripe rust severity, % | | | | Seedlings reaction |
| --- | --- | --- | --- | --- | --- | --- | --- |
|  |  |  | Erzurum | Haymana | Izmir | Mean |  |
| ***Gerek (check 1)*** | | ***greacum*** | ***19*** | ***47*** | ***51*** | ***39*** | ***S*** |
| ***Karahan (check 2)*** | | ***erythrospermum*** | ***6*** | ***34*** | ***24*** | ***21*** | ***S*** |
| **Bread wheat landraces (*Tr. aestivim* sp. *aestivum*)** | | | | | | | |
| No name (Aksaray)109 | | *ferrugineum* | 10 | 1 | 20 | 10 | S |
| Albostan (Nevsehir)30 | | *meridionale* | 10 | 20 | 20 | 17 | S |
| Kobak (Kutahya)152 | | *hostianum* | 10 | 20 | 20 | 17 | R |
| Ari Bugday (Usak)139 | | *greacum* | 5 | 30 | 20 | 18 | S |
| No name (Burdur)164 | | *erytrospermum* | 10 | 30 | 30 | 30 | S |
| Kamci (Nugde)73 | | *pseudo-hostianum* | 5 | 40 | 20 | 22 | S |
| Sahman (Aksaray)119 | | *erytroleucum* | 20 | 40 | 20 | 27 | S |
| No name (Burdur)161 | | *ferrugineum* | 20 | 50 | 20 | 30 | S |
| Kamci (Nigde)71 | | *greacum* | 30 | 40 | 20 | 30 | S |
| Zerun (Sivas)134 | | *delfi* | 40 | 20 | 30 | 30 | S |
| Kamci (Nigde)57 | | *albirubrum* | 50 | 20 | 20 | 30 | S |
| ***Average*** | | ***-*** | ***41*** | ***53*** | ***60*** | ***62*** | ***-*** |
| **Bread wheat landraces (*Tr. aestivim* sp. *aestivum grex compactoidum*)** | | | | | | | |
| Ari Bugday (Usak)141 | | *pseudo-turcicum-compactoides* | 10 | 50 | 20 | 27 | S |
| ***Average*** | | ***-*** | ***39*** | ***96*** | ***71*** | ***68*** | ***-*** |
| **Club wheat landraces (*Tr. aestivim* sp. *compactum*)** | | | | | | | |
| Kobak (Kutahya)154 | | *surchianum* | 20 | 0 | 20 | 13 | R |
| ***Average*** | |  | ***41*** | ***94*** | ***71*** | ***68*** | ***-*** |
| Severity correlation | Erzurum | - | 1.00 | 0.37*** | 0.26** | - | - |
|  | Haymana | - | 0.37*** | 1.00 | 0.43*** | - | - |
