# Supplementary material for: Mining Centuries Old In situ Conserved Turkish Wheat Landraces for Grain Yield and Stripe Rust Resistance Genes
Source: Front Genet. 2016 Nov 18;7:201. doi: 10.3389/fgene.2016.00201 (PMC5114521; doi:10.3389/fgene.2016.00201)
Supplement: Supplementary file 7 [file Table7.DOCX]

| Chromosome | Number of markers | DI | PIC |
| --- | --- | --- | --- |
| 1A | 316 | 0.29 | 0.23 |
| 1B | 387 | 0.27 | 0.22 |
| 1D | 101 | 0.27 | 0.22 |
| 2A | 306 | 0.29 | 0.23 |
| 2B | 467 | 0.29 | 0.23 |
| 2D | 150 | 0.27 | 0.20 |
| 3A | 287 | 0.26 | 0.21 |
| 3B | 399 | 0.29 | 0.23 |
| 3D | 66 | 0.29 | 0.23 |
| 4A | 205 | 0.24 | 0.20 |
| 4B | 113 | 0.24 | 0.20 |
| 4D | 20 | 0.17 | 0.15 |
| 5A | 271 | 0.30 | 0.24 |
| 5B | 430 | 0.29 | 0.23 |
| 5D | 55 | 0.29 | 0.23 |
| 6A | 268 | 0.30 | 0.24 |
| 6B | 382 | 0.26 | 0.21 |
| 6D | 86 | 0.30 | 0.24 |
| 7A | 304 | 0.23 | 0.20 |
| 7B | 278 | 0.30 | 0.24 |
| 7D | 111 | 0.20 | 0.17 |
| A sub-genome | 1957 | 0.27 | 0.22 |
| B sub-genome | 2456 | 0.28 | 0.22 |
| D sub-genome | 589 | 0.26 | 0.21 |

Supp. Table 7 Number of markers on each chromosome and diversity parameters [Nei’s diversity index (DI) and polymorphic information content (PIC)] for each chromosome
