# Supplementary material for: Mining Centuries Old In situ Conserved Turkish Wheat Landraces for Grain Yield and Stripe Rust Resistance Genes
Source: Front Genet. 2016 Nov 18;7:201. doi: 10.3389/fgene.2016.00201 (PMC5114521; doi:10.3389/fgene.2016.00201)
Supplement: Supplementary file 8 [file Table8.DOCX]

Supp. Table 8 Diversity of color + awn groups in Turkish landraces

| Color + awn group | Presence/absence of awn | Glume color | Kernel color | Club/Bread wheat | Botanical variety(s) | Marker-based gne diversity | Marker-based Shannon’s index |
| --- | --- | --- | --- | --- | --- | --- | --- |
| 1 | Yes | Red | Different grain color | Mainly club wheat | Erytroleucum compactoides, Feruginueum compactoides | 0.162 | 0.251 |
| 2 | No | White | White | Mainly club wheat | Albidum-compactoides | 0.039 | 0.063 |
| 3* | Yes | White | Different grain color | Mainly club wheat | Erytrospermum-compactoides, Greacum- compactoides | - | - |
| 4 | No | Red | White grain color | Bread wheat | Albirubrum, Delfi and Pyrotrix | 0.073 | 0.131 |
| 5 | Yes | Red | White grain color | Bread wheat | Mainly Erytroleucum, a few Ferugineum with color+awn group 5 | 0.153 | 0.255 |
| 6* | No | White | Different grain color | Bread wheat | Leucospermum, Velutinum | - | - |
| 7 | Yes | White | Red grain color | Bread wheat | Erytrospermum, Ferugineum with color+awn group 7, Greacum, Meridionale, Pseudo-meridionale, Hostianum, Pseudo hostianum | 0.247 | 0.377 |
| 8 | Awn and awnless | Different glume color | Different grain color | Mainly club wheat | Highly diverse group constuted by Fetissovii, Creticum, Crassiceps, Erynaceum, Rufulum, Humboldtii, Compactum, Rubrum and Surchianum | 0.194 | 0.299 |
| 9 |  |  |  | Bread wheat checks | Gerek and Karahan | 0.176 | 0.246 |

* Gene diversity and Shannon’s index of color + awn groups 3 and 6 were not calculated as they contained only a few accessions and the groups were not found to be genetically different in PCA and STRUCTURE analysis
