# Supplementary material for: Mining Centuries Old In situ Conserved Turkish Wheat Landraces for Grain Yield and Stripe Rust Resistance Genes
Source: Front Genet. 2016 Nov 18;7:201. doi: 10.3389/fgene.2016.00201 (PMC5114521; doi:10.3389/fgene.2016.00201)
Supplement: Supplementary file 10 [file Table10.DOCX]

Supp. Table 10 MTA for APR identified exclusively in club bread wheat

| Marker | Chromosome | Position (cM) | GBS clone ID | Environment | Comment |
| --- | --- | --- | --- | --- | --- |
| M413 | 3AS | 89.8 | 1125482 | Erzurum, Izmir | Novel |
| M670 | 5BS | 55.7 | 1012837 | Erzurum | Known (QRYr5B.1) |
| M755 | 2DL | 116.2 | 1077498 | Izmir | Known (QRYr2D.2) |
