# Supplementary material for: Mining Centuries Old In situ Conserved Turkish Wheat Landraces for Grain Yield and Stripe Rust Resistance Genes
Source: Front Genet. 2016 Nov 18;7:201. doi: 10.3389/fgene.2016.00201 (PMC5114521; doi:10.3389/fgene.2016.00201)
Supplement: Supplementary file 11 [file Table11.DOCX]

Supp. Table 11 Epistatic interactions for grain yield and yield components among markers with main effects in different environments

| Environment | Trait | Locus combination | Marker alleles involved in epistasis | Percentage variation explained by interaction (%) |
| --- | --- | --- | --- | --- |
| Hamidiye | GY | 2 | M4844(BB) M2670(AA) | 1.9 |
|  |  | 3 | M6113(BB) M5746(BB) M3062(AA) | 2.6 |
|  |  | 3 | M1830(AA) M4844(BB) M2670(AA) | 2.1 |
|  | TSPS | 2 | M4457(BB) M7537(BB) | 8.4 |
|  |  | 3 | M6968(AA) M1472(BB) M7537(AA) | 7.9 |
|  |  | 3 | M4457(BB) M7537(BB) M2076(BB) | 8.7 |
|  |  | 3 | M4457(BB) M7537(BB) M4885(AA) | 8.8 |
|  |  | 3 | M1830(AA) M2076(BB) M1444(AA) | 7.2 |
|  | TKW | 2 | M4831(BB) M3891(AA) | 6.5 |
|  |  | 3 | M4831(BB) M3891(AA) M3037(AA) | 6.2 |
|  | SW | 2 | - | - |
|  |  | 3 | - | - |
|  | SL | 2 | M5514(AA) M3345(AA) | 3.4 |
|  |  | 2 | M4457(AA) M3345(BB) | 3.1 |
|  |  | 2 | M3345(AA) M2420(AA) | 3.1 |
|  |  | 3 | - | - |
|  | SHI | 2 | - | - |
|  |  | 3 | M4457(AA) M7822(BB) M3062(AA) | 3.0 |
|  |  | 3 | M1806(AA) M7822(BB) M3062(AA) | 3.0 |
|  | SD | 2 | M1678(AA) M3345(AA) | 3.7 |
|  |  | 3 | - | - |
|  | GWPS | 2 | M4457(AA) M4506(BB) | 4.2 |
|  |  | 2 | M2379(AA) M4506(BB) | 3.5 |
|  |  | 2 | M7537(AA) M4506(BB | 4.7 |
|  |  | 3 | - | - |
|  | GNPS | 2 | M6758(BB) M2762(AA) | 1.0 |
|  |  | 3 | M6758(BB) M2762(AA) M5062(AA) | 1.5 |
|  | FI | 2 | M7822(BB) M3062(AA) | 2.9 |
|  |  | 3 | M1394(BB) M6113(AA) M2242(AA) | 2.5 |
|  |  | 3 | M1394(BB) M7822(BB) M3062(AA) | 3.0 |
|  |  | 3 | M5514(AA) M7822(BB) M3062(AA) | 3.1 |
|  |  | 3 | M6113(BB) M7822(BB) M3062(AA) | 3.0 |
|  |  | 3 | M1480(AA) M7822(BB) M3062(AA) | 2.9 |
|  |  | 3 | M7822(BB) M3062(AA) M2077(AA) | 3.0 |
|  | FSPS | 2 | - | - |
|  |  | 3 | M679(AA) M2762(AA) M7537(BB) | 3.9 |
|  |  | 3 | M4457(AA) M2762(BB) M7537(AA) | 3.4 |
|  |  | 3 | M2762(AA) M7537(BB) M2076(BB) | 3.4 |
| Environment | Trait | Locus combination | Marker alleles involved in epistasis | Percentage variation explained by interaction (%) |
| Hamidiye | CW | 2 | M8412(AA) M3080(BB) | 12.9 |
|  |  | 2 | M8412(AA) M3080(AA) | 12.6 |
|  |  | 2 | M2242(AA) M3080(AA) | 9.1 |
|  |  | 2 | M3080(BB) M4506(AA) | 9.8 |
|  |  | 3 | M8412(AA) M2242(AA) M3080(BB) | 14.3 |
|  |  | 3 | M8412(AA) M2242(AA) M3080(AA) | 15.4 |
|  |  | 3 | M8412(AA) M3080(BB) M7537(AA) | 17.5 |
|  |  | 3 | M8412(AA) M3080(AA) M7537(AA) | 18.1 |
|  |  | 3 | M3080(BB) M7537(AA) M4506(AA) | 14.2 |
|  | CPS | 2 | M3080(BB) M4506(AA) | 13.9 |
|  |  | 3 | M2242(AA) M3080(BB) M4506(AA) | 15.2 |
|  |  | 3 | M3080(BB) M1913(AA) M4506(AA) | 14.8 |
|  | PH | 2 | M1850(AA) M2057(BB) | 4.1 |
|  |  | 3 | - | - |
| I. Cumra | GY | 2 | M3750(AA) M3295(AA) | 7.7 |
|  |  | 2 | M3295(AA) M3062(BB) | 8.3 |
|  |  | 3 | M3750(AA) M3295(AA) M3037(AA) | 8.7 |
|  |  | 3 | M4457(AA) M3750(AA) M3295(AA) | 8.0 |
|  |  | 3 | M3750(AA) M3295(AA) M3062(BB) | 8.6 |
|  |  | 3 | M3295(AA) M3037(AA) M3062(BB) | 8.4 |
|  |  | 3 | M3295(AA) M5062(AA) M3062(BB) | 7.9 |
|  | Photo u | 2 | M6200(BB) M2742(AA) | 2.7 |
|  |  | 3 | M6200(BB) M2742(AA) M3880(AA) | 3.5 |
|  | TSPS | 2 | M7253(BB) M8436(BB) | 11.5 |
|  |  | 2 | M7253(BB) M3062(BB) | 10.1 |
|  |  | 3 | M7253(BB) M3295(AA) M3062(BB) | 19.6 |
|  | TKW | 2 | M3295(AA) M3966(AA) | 14.1 |
|  |  | 3 | M5625(AA) M3295(AA) M3966(AA) | 26.8 |
|  |  | 3 | M4278(BB) M4166(BB) M3966(AA) | 24.4 |
|  |  | 3 | M4278(BB) M3295(AA) M3966(AA) | 24.4 |
|  |  | 3 | M3891(AA) M3295(AA) M3966(AA) | 24.8 |
|  |  | 3 | M4166(BB) M3295(AA) M3966(AA) | 24.2 |
|  |  | 3 | M3295(AA) M3037(AA) M3966(AA) | 24.8 |
|  | SW | 2 | M5074(AA) M7253(BB) | 10.6 |
|  |  | 3 | M613(AA) M5074(AA) M1820(AA) | 10.3 |
|  |  | 3 | M5074(AA) M1820(AA) M7253(BB) | 12.3 |
|  |  | 3 | M5074(AA) M1610(AA) M7253(BB) | 12.6 |
|  | SL | 2 | M3771(AA) M7881(BB) | 9.4 |
|  |  | 3 | M3771(AA) M7881(BB) M3062(BB) | 10.6 |
|  | SHI | 2 | M2762(BB) M7822(AA) | 7.5 |
|  |  | 3 | M5625(AA) M2762(BB) M7822(AA) | 15.9 |
| Environment | Trait | Locus combination | Marker alleles involved in epistasis | Percentage variation explained by interaction (%) |
| I Cumra |  | 3 | M3036(BB) M2762(BB) M7822(AA) | 15.8 |
|  | SD | 2 | M8578(BB) M3966(BB) | 13.1 |
|  |  | 3 | M7881(BB) M8578(BB) M3966(BB) | 15.2 |
|  | GWPS | 2 | M5074(AA) M1820(AA) | 4.8 |
|  | GWPS | 3 | M613(AA) M5074(AA) M1820(AA) | 8.8 |
|  | GNPS | 2 | M1820(BB) M8096(AA) | 7.7 |
|  |  | 3 | - | - |
|  | FI | 2 | M6721(AA) M7822(AA) | 10.4 |
|  |  | 3 | M5625(AA) M6721(AA) M7822(AA) | 14.7 |
|  | FSPS | 2 | - |  |
|  |  | 3 | - |  |
|  | CW | 2 | - | - |
|  |  | 3 | M613(AA) M5074(AA) M1820(AA) | 11.7 |
|  |  | 3 | M5074(AA) M1820(AA) M2976(AA) | 11.9 |
|  |  | 3 | M5074(AA) M1610(AA) M2976(AA) | 12.1 |
|  | CPS | 2 | M5150(AA) M3891(BB) | 7.7 |
|  |  | 2 | M3891(BB) M2976(BB) | 6.9 |
|  |  | 2 | M3891(BB) M4465(AA) | 7.5 |
|  |  | 3 | M5150(AA) M3891(BB) M2976(BB) | 7.7 |
|  |  | 3 | M5150(AA) M3891(BB) M4465(AA) | 8.8 |
|  |  | 3 | M5150(AA) M3891(AA) M4465(AA) | 8.3 |
|  |  | 3 | M8086(AA) M7602(BB) M3891(AA) | 10.0 |
|  |  | 3 | M3891(BB) M5954(AA) M4465(AA) | 6.6 |
|  |  | 3 | M3891(BB) M2976(BB) M4465(AA) | 7.8 |
|  | PH | 2 | M4682(AA) M3314(BB) | 18.2 |
|  |  | 3 | M4682(AA) M3314(BB) M1514(AA) | 20.5 |
|  | DH | 2 | - | - |
|  |  | 3 | - | - |
| Erzurum | GY | 2 | M3056(AA) M4506(BB) | 4.1 |
|  |  | 3 | M3056(AA) M3295(BB) M4506(BB) | 4.0 |
|  | PH | 2 | M3314(BB) M1514(AA) | 7.7 |
|  |  | 3 | M4682(AA) M3314(BB) M1514(AA) | 7.8 |
|  | DH | 2 | M2943(BB) M8096(BB) | 4.5 |
|  |  | 3 | M2943(BB) M1269(AA) M8096(BB) | 4.4 |
