# Supplementary material for: Mining Centuries Old In situ Conserved Turkish Wheat Landraces for Grain Yield and Stripe Rust Resistance Genes
Source: Front Genet. 2016 Nov 18;7:201. doi: 10.3389/fgene.2016.00201 (PMC5114521; doi:10.3389/fgene.2016.00201)
Supplement: Supplementary file 12 [file Table12.DOCX]

| Environment | Genes  included in model  (+/-) | Locus combination | Marker and gene alleles involved in epistasis | Percentage variation explained by interaction (%) |
| --- | --- | --- | --- | --- |
| Erzurum | - | 2 | - | - |
|  | - | 3 | M6398(BB) M6872(BB) M1550(AA) | 9.7 |
|  | + | 2 | - | - |
|  | + | 3 | - | - |
| Haymana | - | 2 | M6398(BB) M4676(BB) | 9.0 |
|  | - | 3 | - | - |
|  | + | 2 | - | - |
|  | + | 3 | - | - |
| Izmir | - | 2 | - |  |
|  | - | 3 | M6160(AA) M6398(BB) M6287(AA) | 7.0 |
|  | + | 2 | - | - |
|  | + | 3 | - | - |
| Combined environments | - | 2 | M6660 (BB) M6398(BB) | 7.9 |
|  | - | 3 | M6872 (BB) M6660 (BB) M6398(BB) | 9.5 |

Supp. Table 12 Epistatic interactions for stripe rust resistance among markers with main effects in different environments

Highlighted marker represents main epicentric locus interacting with other loci
