# Supplementary material for: Mining Centuries Old In situ Conserved Turkish Wheat Landraces for Grain Yield and Stripe Rust Resistance Genes
Source: Front Genet. 2016 Nov 18;7:201. doi: 10.3389/fgene.2016.00201 (PMC5114521; doi:10.3389/fgene.2016.00201)
Supplement: Supplementary file 13 [file Table13.DOCX]

Supp. Table 13 QTL for GY, yield stability and spike productivity traits identified in the study

| Chromosome | QTL | Position (cM) | Tagged marker(s) | GBS clone ID | Comment* |
| --- | --- | --- | --- | --- | --- |
| 1B | QTL1B.1 | 61.58 | M4506 | 997799 | Novel |
|  | QTL1B.2 | 104.00 | M1913 | 2275550 | Known (MQTL4) |
|  | QTL1B.3 | 177.18 | M3062 | 1260851 | Novel |
|  | QTL1B.4 | 336.91 | M5062 | 3025163 | Novel |
| 2A | QTL2A.1 | 113.00 | M8096 | 1161563 | - |
|  | QTL2A.2 | 127.65-129.43 | M3037, M6456 | 1215845, 1229432 | - |
|  | QTL2A.3 | 183.75 | M4465 | 2372250 | - |
| 2B | QTL2B.1 | 3.93 | M2916 | 1075469 | Novel |
|  | QTL2B.2 | 119.56 | M3295 | 1105759 | Known (MQTL11) |
|  | QTL2B.3 | 150.25-159.97 | M6463, M8899,  M1292 | 1120855, 3023409,  1120855 | Novel |
| 4A | QTL4A.1 | 160.24 | M7602 | 1093756 | Novel |
|  | QTL4A.2 | 177.34 | M4831 | 1217741 | Known (MQTL 32) |
|  | QTL4A.3 | 227.27 | M3056 | 1237142 | Novel |
| 4B | QTL4B.1 | 86.8-93.5 | M4439, M1820 | 1696122, 1209504 | Known (MQTL33) |
| 5B | QTL5B.1 | 55.28-59.46 | M7537, M2670 | 1212937, 1229558 | Novel |
|  | QTL5B.2 | 153.16-153.69 | M1830, M4844 | 1216209, 1255792 | Known (Bordes et al. 2014) |
|  | QTL5B.3 | 270.59-272.43 | M4827, M5746, M2762 | 1202879, 1201842,  3026522 | Novel |
| 6A | QTL6A.1 | 52.45 | M4457 | 2264705 | Novel |
| 6B | QTL6B.1 | 4.60-12.36 | M2242, M6113 | 1216037, 1221034 | Novel |
| 7A | QTL7A.1 | 144.75 | M8412 | 2306534 | Novel |

*QTLs categorized as known or novel based on metaQTL (MQTL) reported by Zhang et al. (2010) and studies published afterwards
