# Supplementary material for: Mining Centuries Old In situ Conserved Turkish Wheat Landraces for Grain Yield and Stripe Rust Resistance Genes
Source: Front Genet. 2016 Nov 18;7:201. doi: 10.3389/fgene.2016.00201 (PMC5114521; doi:10.3389/fgene.2016.00201)
Supplement: Supplementary file 14 [file Image1.PDF]

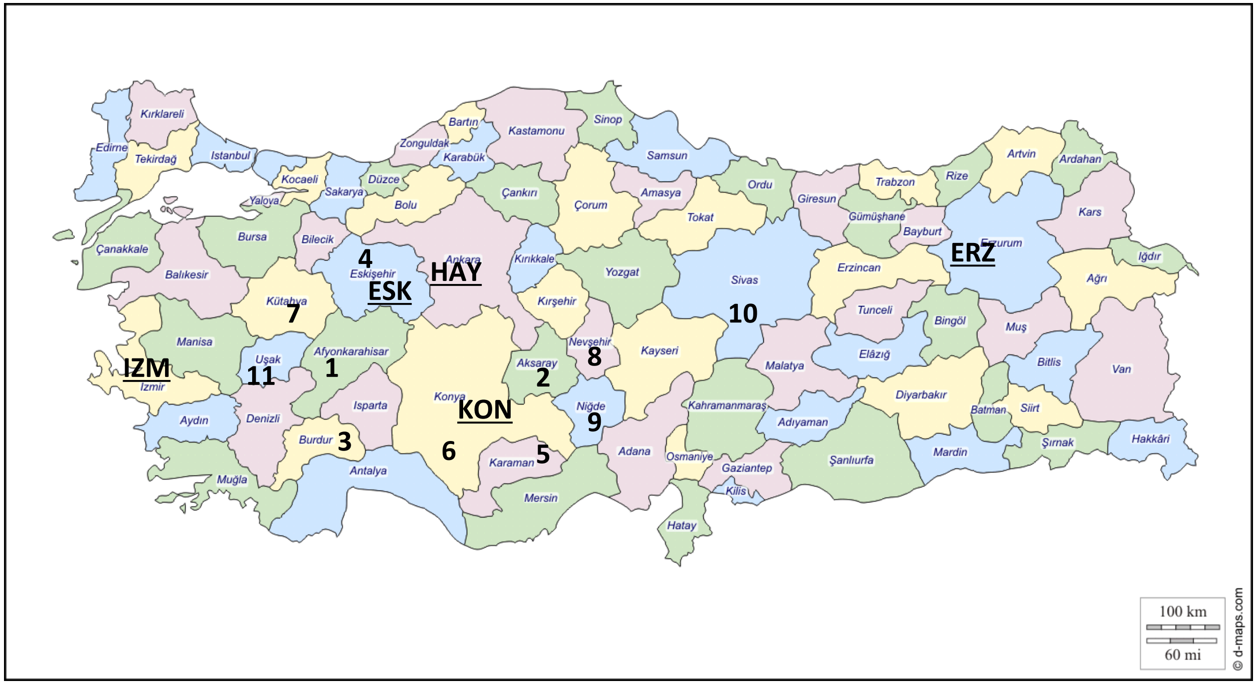

Supp. Figure 1. Origin of wheat landraces used in the study: 1. Afyonkarahisar (3 lines, 3 morphotypes). 2. Aksaray (60 lines, 20 morphotypes). 3. Burdur (7 lines, 3 morphotypes). 4. Eskisehir (1 line, 1 morphotype). 5. Karaman (12 lines, 9 morphotypes). 7. Kutahya (7 lines, 6 morphotypes). 8. Nevsehir (12 line, 7 morphotypes). 9. Nigde (24 lines, 13 morphotypes). 10. Sivas (1 line, 1 morphotype). 11. Usak (13 line, 9 morphotypes). Testing sites: ESK – Eskisheir, KON – Konya, ERZ – Erzurum, HAY – Haymana, IZM – Izmir.
