# Supplementary material for: Mining Centuries Old In situ Conserved Turkish Wheat Landraces for Grain Yield and Stripe Rust Resistance Genes
Source: Front Genet. 2016 Nov 18;7:201. doi: 10.3389/fgene.2016.00201 (PMC5114521; doi:10.3389/fgene.2016.00201)
Supplement: Supplementary file 15 [file Image2.PDF]

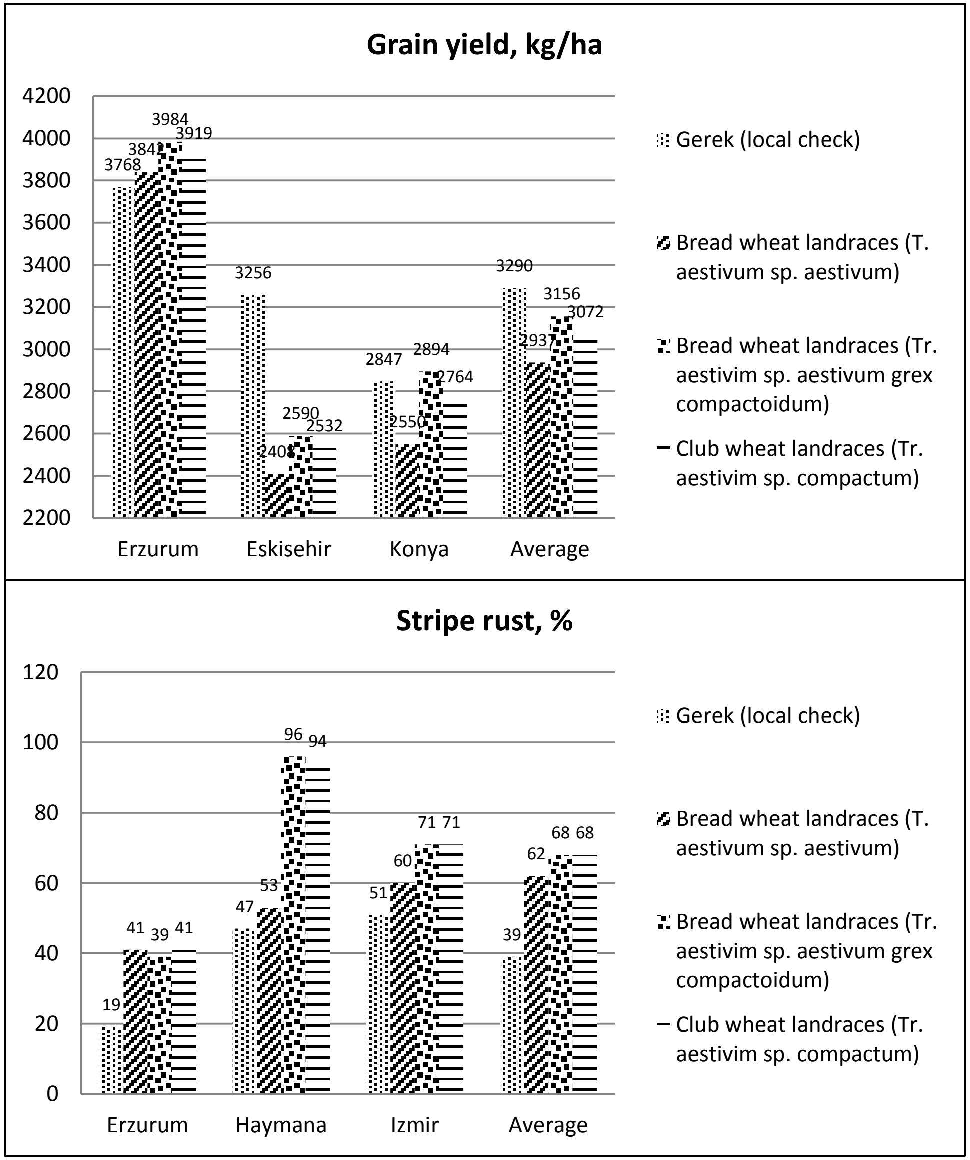

Supp. Figure 2 Average grain yield (kg/ha) and stripe rust severity (%) of wheat landraces sub-species evaluated in Turkey in 2013.
