# Supplementary material for: Mining Centuries Old In situ Conserved Turkish Wheat Landraces for Grain Yield and Stripe Rust Resistance Genes
Source: Front Genet. 2016 Nov 18;7:201. doi: 10.3389/fgene.2016.00201 (PMC5114521; doi:10.3389/fgene.2016.00201)
Supplement: Supplementary file 16 [file Image3.PDF]

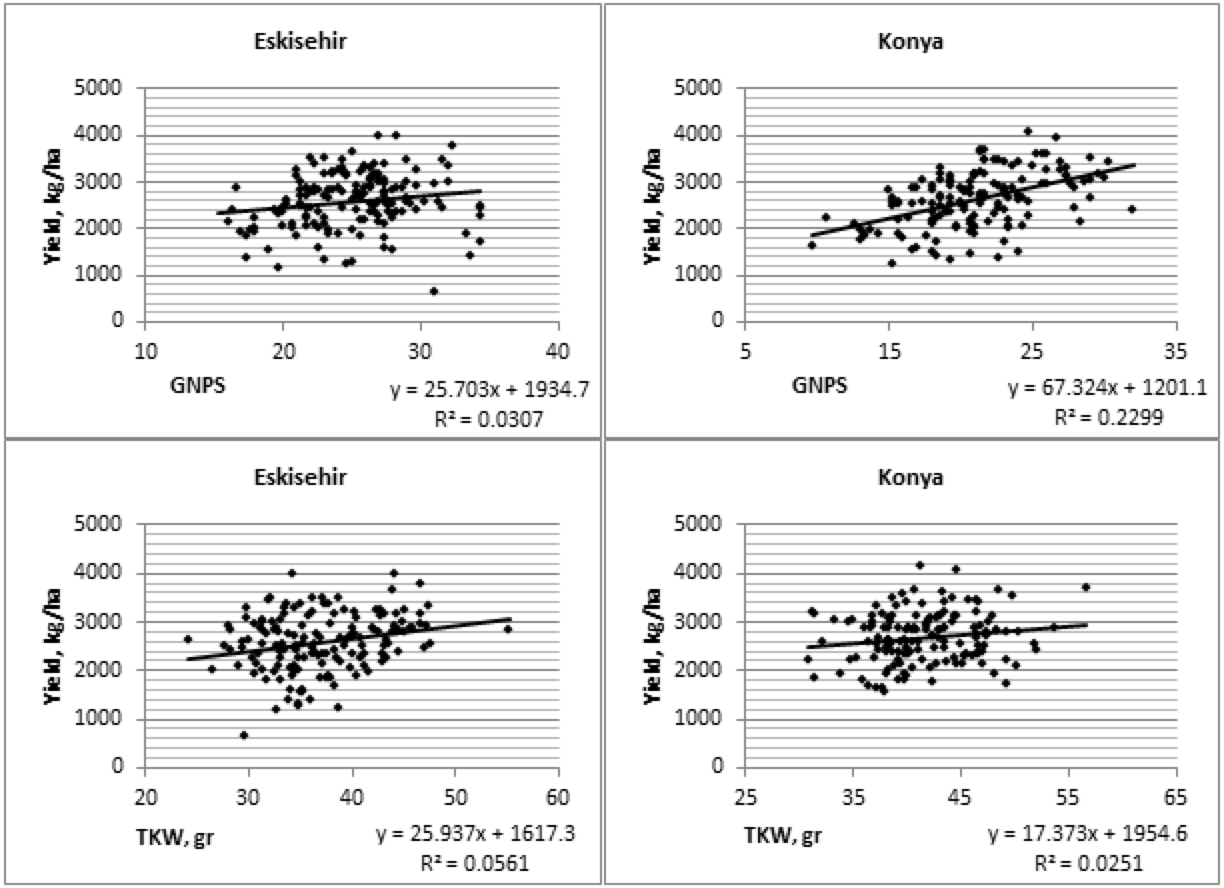

Supp. Figure 3. Relationship between grain yield a, grain number of per spike (GNPS) and thousand kernel weight (TKW) in a set of selections from wheat landraces tested in Eskisehir and Konya
