# Supplementary figures and images for: Mining Centuries Old In situ Conserved Turkish Wheat Landraces for Grain Yield and Stripe Rust Resistance Genes
Source: Front Genet. 2016 Nov 18;7:201. doi: 10.3389/fgene.2016.00201 (PMC5114521; doi:10.3389/fgene.2016.00201)

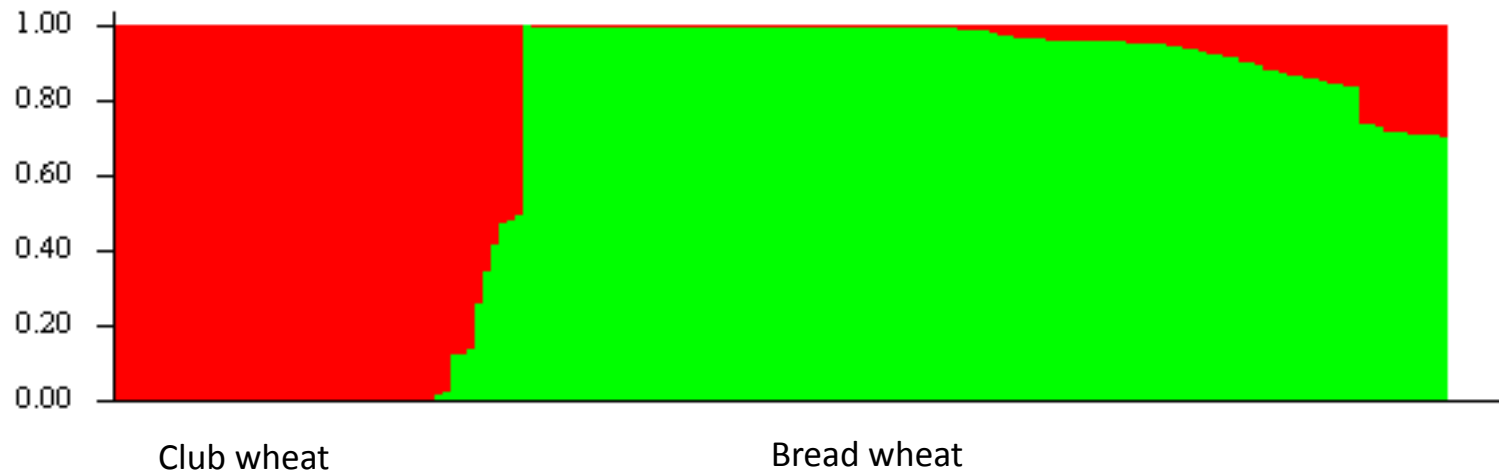

Supp. Figure 4 Population structure of Turkish landraces at K = 2

Supplement: Supplementary file 17 [file Image4.PDF]

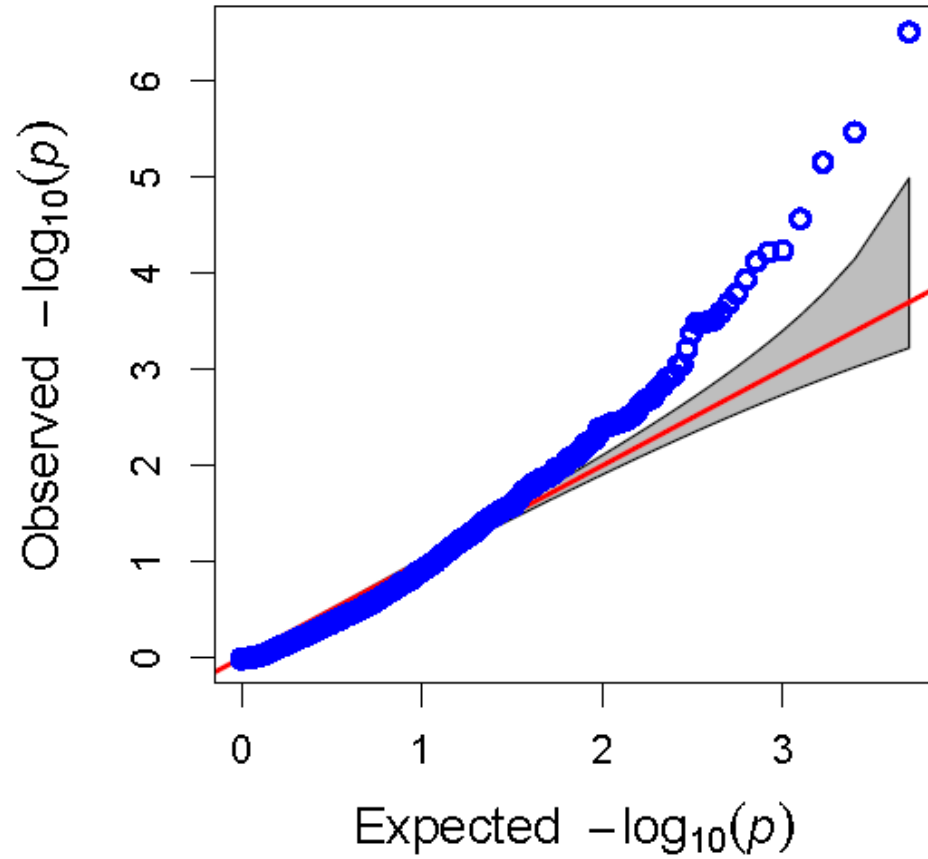

**Supplementary Figure 8** QQ plots of stripe rust resistance

Supplement: Supplementary file 21 [file Image8.PDF]
