# Supplementary material for: Mining Centuries Old In situ Conserved Turkish Wheat Landraces for Grain Yield and Stripe Rust Resistance Genes
Source: Front Genet. 2016 Nov 18;7:201. doi: 10.3389/fgene.2016.00201 (PMC5114521; doi:10.3389/fgene.2016.00201)
Supplement: Supplementary file 18 [file Image5.PDF]

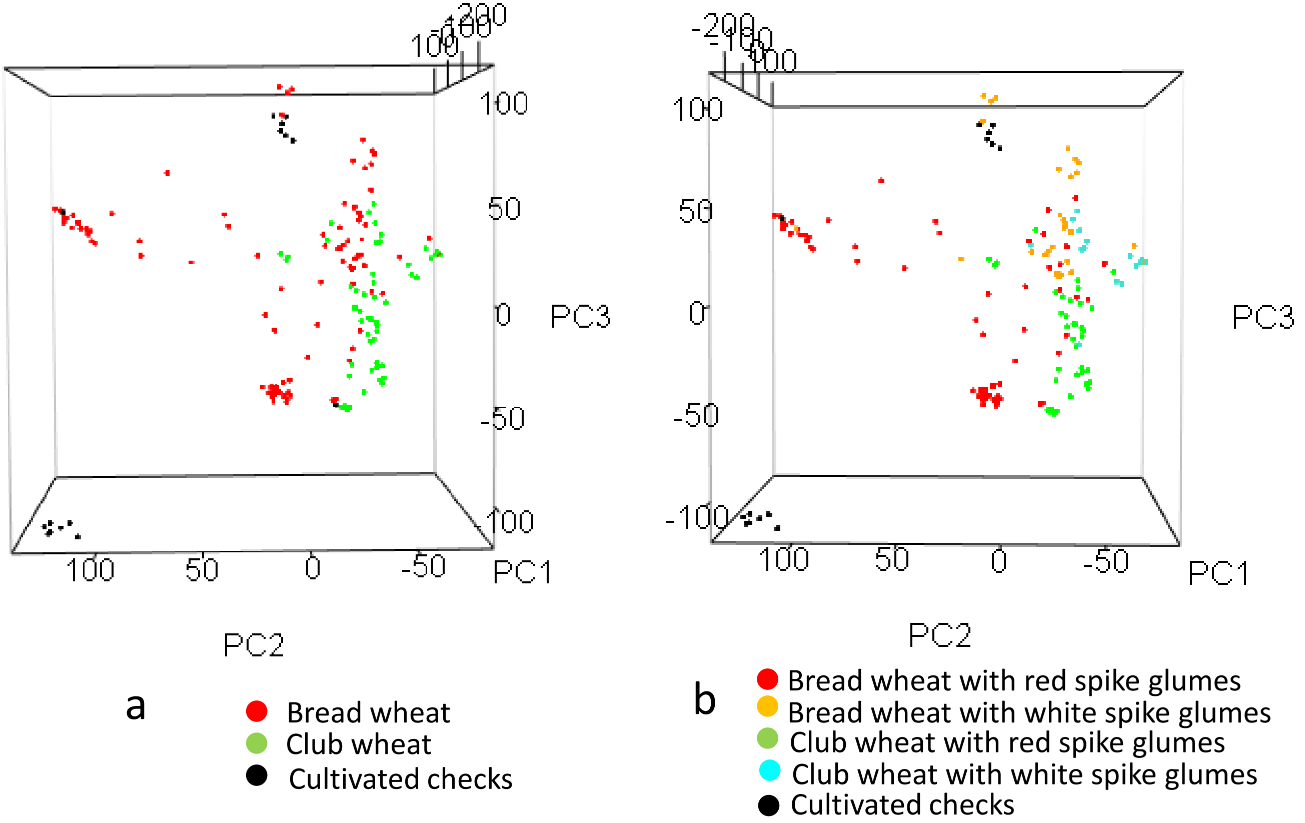

Supp. Figure 5 Principal component analysis plot with three axes showing discrimination between two subspecies (a) and red and white spike glume groups of the two subspecies (b)
