# Supplementary material for: Mining Centuries Old In situ Conserved Turkish Wheat Landraces for Grain Yield and Stripe Rust Resistance Genes
Source: Front Genet. 2016 Nov 18;7:201. doi: 10.3389/fgene.2016.00201 (PMC5114521; doi:10.3389/fgene.2016.00201)
Supplement: Supplementary file 19 [file Image6.PDF]

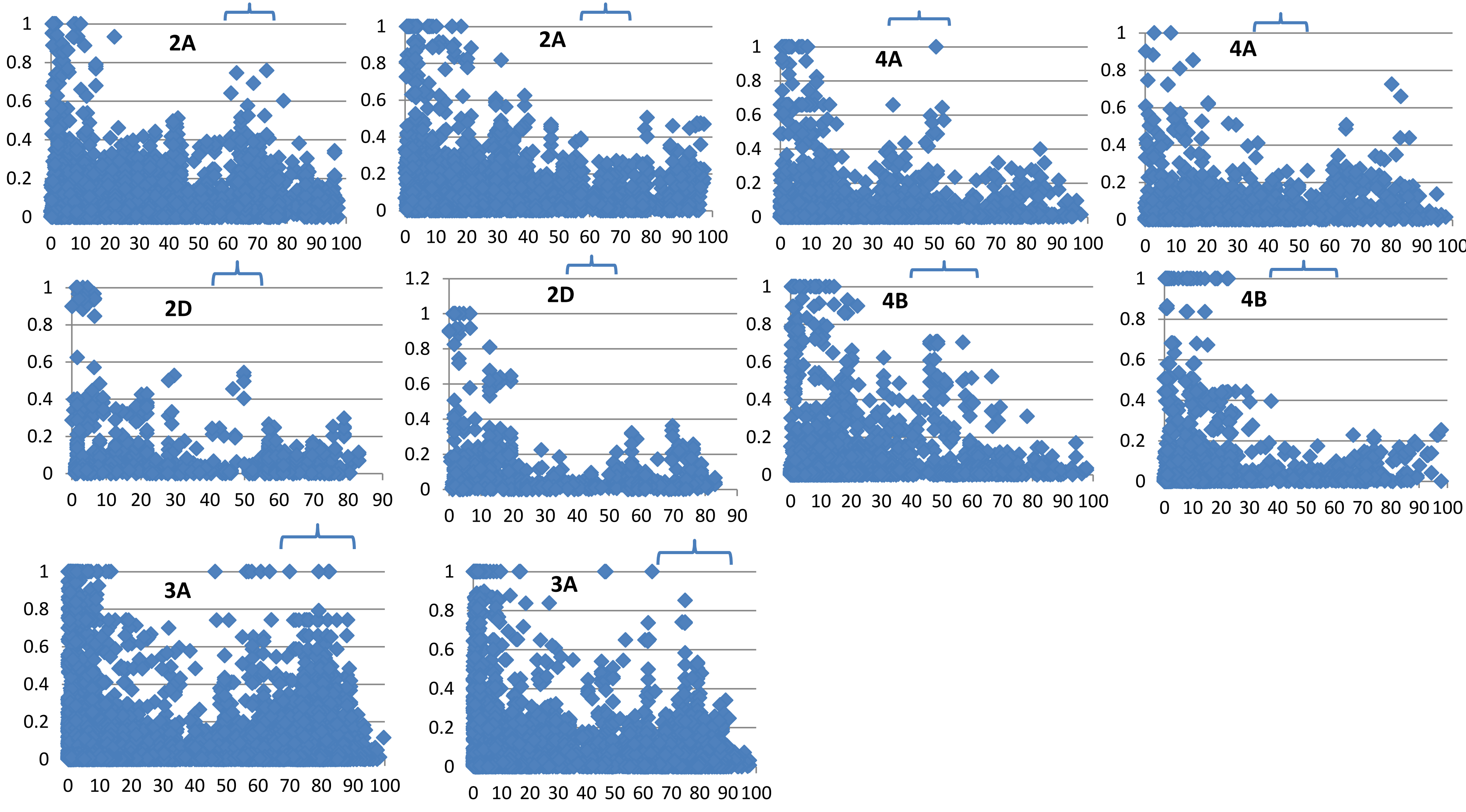

Supp. Figure 6 Linkage disequilibrium (LD) of markers on different chromosomes in BW (left) and CW (right). Values at Y-axis represent squared correlation coefficient r^2 and values at X-axis represent genetic distance in cM. The genomic regions are arrowed where the two subspecies show contrasting LD among markers
