# Supplementary material for: Mining Centuries Old In situ Conserved Turkish Wheat Landraces for Grain Yield and Stripe Rust Resistance Genes
Source: Front Genet. 2016 Nov 18;7:201. doi: 10.3389/fgene.2016.00201 (PMC5114521; doi:10.3389/fgene.2016.00201)
Supplement: Supplementary file 20 [file Image7.PDF]

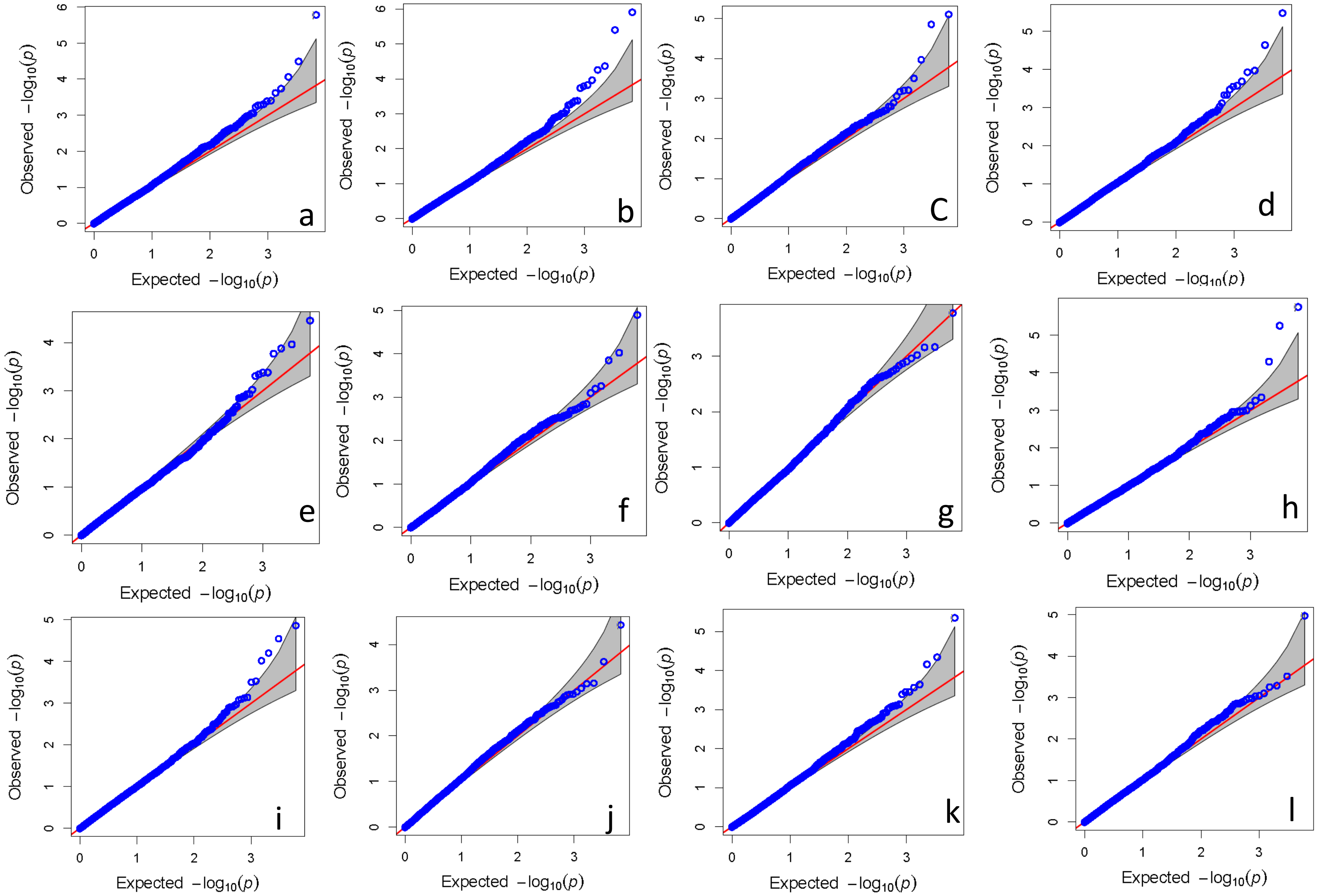

Supplementary Figure 7 QQ plots of grain yield (a), total spikelets per spike (b), thousand kernel weight (c), spike weight (d), spike length (e), spike harvest index (f), spike density (g), grain weight per spike (h), fertility index (i), fertile spikelets per spike (j), plant height (k) and days to heading (l)
